# Effect of Storage Time on the Physical, Chemical, and Rheological Properties of Blueberry Jam: Experimental Measurements and Artificial Neural Network Simulation

**DOI:** 10.3390/foods12152853

**Published:** 2023-07-27

**Authors:** Daniela Helena Pelegrine Guimarães, Ana Lúcia Gabas Ferreira, Pedro Felipe Arce

**Affiliations:** 1 Department of Chemical Engineering, Engineering School of Lorena, University of São Paulo, Lorena 12602-810, SP, Brazil; dhguima@usp.br (D.H.P.G.); parce@usp.br (P.F.A.); 2 Department of Basic and Environmental Sciences, Engineering School of Lorena, University of São Paulo, Lorena 12602-810, SP, Brazil

**Keywords:** blueberry jam, storage, rheological properties, artificial neural networks, molecular descriptors

## Abstract

The present work aimed to develop different formulations of blueberry jam (traditional and light) made from rabbiteye fruits (Powder Blue and Climax varieties) and then analyze the influence of storage on their physicochemical and rheological properties at different times: (i) zero time (i.e., freshly processed), (ii) after 30 days, (iii) after 90 days and (iv) after 120 days. The influence of storage time on these properties of the jams was analyzed using statistical analysis (ANOVA and Tukey test) and regression. The physical, chemical and rheological properties were predicted by mathematical simulation using independent variables composed of molecular descriptors + SMILES codes. It also used time (days), % water, % citric acid, % glucose, % sucrose, % anthocyanin, % HM pectin, % LM pectin, % xanthan gum, pH and acidity (%), as independent variables. Several architectures of three and four layers for learning were tested, encompassing testing and prediction steps in order to predict the dependent variables of hardness, water activity, and adhesiveness. According to the results, higher sucrose concentrations and longer cooking times showed greater anthocyanin instability in products made with HM pectin (i.e., in traditional products). In the same way, there was no influence of the storage time on soluble solids content in light jellies (made with LM pectin). Regarding the rheological properties, it was noted that time influenced the hardness of the jellies, except for the traditional formulation with pectin extracted from the passion fruit peel (highly hydrated). However, adhesiveness was influenced by time in all products. The lowest deviations for the dependent variables were obtained, finding the optimal configuration of 10-30-10-3 architecture. The lowest deviations for the dependent variables were obtained, finding the optimal configuration of 10-30-10-3 architecture.

## 1. Introduction

The blueberry is a fruit that stands out in the world fruit crop due to its excellent nutritional value. Worldwide, it is considered the fruit of longevity since its anthocyanin content is high, which prevents several degenerative diseases (due to the high antioxidant capacity of the anthocyanins) and, thus, contributes to a healthier life [1,2,3,4]. Despite this, anthocyanins are phenolic compounds, which can have negative impacts on the sensory quality of the fruit, impairing its consumer acceptance. In this context, the manufacture of jams is an alternative to soften the astringent taste [5,6] and, in addition, the production of fruit jellies is considered a leading industrially important product, especially in European countries, such as England, which assumes a prominent role both in terms of consumption and quality [7,8].

The literature defines fruit jam as the product obtained by cooking fruit (whole, in pieces or fruit juice) with sugar and water and concentrated until it achieves a gelatinous consistency. It cannot be artificially colored or flavored, but the addition of acidulants and pectin is tolerated, if necessary, to compensate for any deficiency in the natural acidity and/or pectin content [8,9].

Pectin, used mainly in the food industry as a gelling agent in jellies and jams, is classified according to the degree of esterification (DE) as low (LM, <50% DE) and high (HM, >50% DE) methoxy. LM pectin is often used in low sugar jams and is obtained by controlled de-esterification of HM pectin under acidic or alkaline conditions. However, pectin processing (e.g., extraction, heat, or pH condition) and functional group distribution vary between factories. Pectin is an important component in texture variation, which represents a critical factor for food acceptability [10].

Even industrialized foods remain susceptible to biological activities evidenced by variations (which can be microbiological, physical, chemical, and even enzymatic), which can result in loss of nutrients, as well as physical, chemical (pH, acidity), and rheological (texture and viscosity) changes in properties [11,12]. One of the main quality attributes that influences the acceptability of jam is texture, which influences its appearance, flavor, and sensory impressions (gustatory and tactile) [13,14]. That is, the consistency of a jam must maintain its semisolid state when removed from its flask, with a smooth texture and without resistance to cutting [15,16].

Texture is defined as the manifestation of the rheological properties of the material, considered an important attribute of food, considering that it affects the process, storage, handling, and acceptance of the product by the consumer [17]. Knowledge of the rheological properties of semisolid foods such as jellies is important for process design, quality control and new product development [18].

Previous research reports the main factors responsible for the rheological behavior of fruit-derived products as the type of fruit, temperature, and solid content such as sugars, pectins and fibers [19].

Broomes and Badrie [20] reported an increase in gel firmness and a decrease in jelly acceptability the higher the added LM pectin content when investigating the effects of adding low methoxyl (LM) pectin on the physical and sensory properties of light jellies. The authors mentioned that all sensory attributes (color, odor, appearance, flavor, texture and general acceptability) were significantly affected by the presence of LM pectin.

In comparison, Basu and Shivhare [21], when investigating the effects of adding sorbitol on the rheological and sensory properties of mango jam, found that increasing the content of this sweetener resulted in a decrease in hardness and an increase in the sensory evaluation of spreadability.

Despite the availability of some studies involving the stability of phenolic compounds in blueberry jams [22,23,24], there are few studies on the effect of storage time on the rheological properties of this type of product.

In general, the properties can be predicted through a mathematical model. There are several methods for identifying a great model that can predict such properties. One successful method in the academic environment is artificial neural networks (ANN). In this context, technical information provided to the ANNs can be obtained from specific properties of the involved substances and from the so-called molecular descriptors.

**Figure 1:**
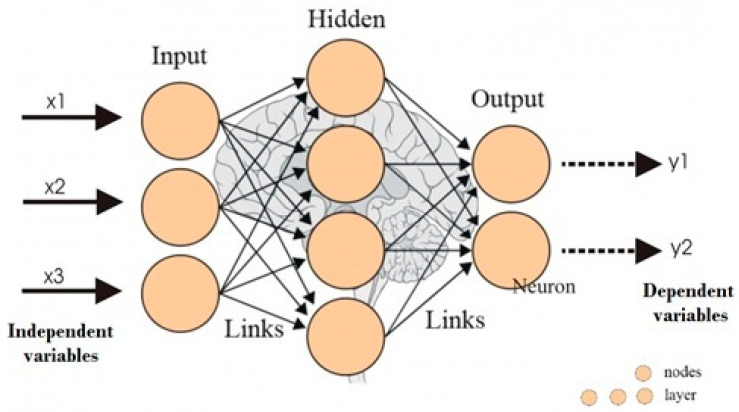
Structure of an artificial neural network (modified from Roy [27]).

An artificial neural network is a system or information-processing paradigm, composed of highly interconnected processing elements or “neurons”, called artificial (nodes), that work to solve specific problems. It is inspired from the biological nervous system, similar to how the brain processes information. In engineering, they greatly resemble human neurons and are also interconnected, just as their human counterparts (Figure 1). These ensembles are arranged into interlinked layers along specified architectures. The most common for the engineering applications is multilayer perception (MLP), which is a feed forward neural network. It consists of input and output layers with hidden layers in between. Each layer has several artificial neurons. The outputs from the input layer are fed to hidden nodes. Input nodes receive some type of information (stimulus) from the outside or other neighboring neurons and process it by sending a hidden link and then the output to neighboring neurons through their related links [25,26].

The field of molecular descriptors is based on the mass of different theories such as algebra, graph theory, information theory, computational chemistry, theories of organic reactivity and physical chemistry. The molecular descriptor is a logic and mathematical procedure transforming chemical information encoded within a symbolic representation of a molecule into a useful number (such as code SMILES) or the result of some standardized experiment. They are fundamental tools used in several areas such as chemistry, pharmaceutical sciences, environmental protection policy, health research, quality control, etc. [28,29].

Molecular descriptors consider the invariance with respect to labeling and numbering of the molecule atoms and to the molecule rototranslation, an unambiguous algorithmically computable definition, and values in a suitable numerical range for the set of molecules. All of them consider the connectivity of atoms in molecules, molecular size, shape, atom distributions, number of atoms, bond count, atom type, ring count, and molecular weight, etc. Thus, they are called topological indexes, geometrical, constitutional, and thermodynamic descriptors, etc., defining the chemical structures [30].

Dragon 7.0 [31] is one of the most important computer programs used to calculate molecular descriptors of the properties of compounds. Dragon calculates the molecular descriptors and fingerprints for more than 5000 molecular descriptors. They are divided into 30 logical blocks, each one divided into subblocks to allow easy retrieval of molecular descriptors. In this work, it is used to obtain the molecular descriptors of five components of blueberry jam (water, citric acid, glucose, sucrose, and anthocyanin). Dragon requires technical information from molecular structure files for calculating the molecular descriptors. These structure files are previously generated by other specific chemical drawing programs. The SMILES format file (.smi) is the most used to obtain the molecular descriptors.

The combination of artificial neural networks (ANN) and molecular descriptors (MD) is a technique recently used for the prediction of physical, chemical, and thermodynamic properties of pure fluids and solutions or mixtures. The authors have already used this technique in several research studies [32,33]. SMILES (Simplified Molecular Input Line Entry System) is a specific, easy, and flexible chemical notation to represent a chemical structure in a way that can be interpreted by the Dragon [34,35]. The SMILES notation requires that the user learns a set of rules. SMILES is used to translate a chemical’s three-dimensional structure (atoms, bonds, aromatic and nonaromatic rings, stereoisomers, isotopes, etc.) into a type of symbol string that is easily understood by the computer software. Other computer programs are available to translate a chemical structure into SMILES codes [36,37].

The present study includes four blueberry jam formulations based on the Climax and Powder Blue varieties. They were prepared as conventional and light jams. High methoxyl pectin (HM) and low methoxyl (LM) pectin were used for gelling and the products were analyzed according to their physical and chemical properties, water activity and texture properties (hardness and adhesiveness), immediately after processing and after 30, 90 and 120 days to analyze the storage effect on jam characteristics. Considering the need for studies that correlate storage time with changes in physical and chemical properties, this study evaluates the influence of storage time on these properties of traditional and light blueberry jam formulations (Climax and Powder Blue). Experimental data were submitted to the mathematical simulation. This approach used artificial neural networks, with which the study was performed based on several configurations to find the optimal configuration. Independent variables assumed in this work were: time (days), % water, % citric acid, % glucose, % sucrose, % anthocyanin, % HM pectin, % LM pectin, % xanthan gum, pH and acidity (%). The jelly molecule is complex, for this reason it was necessary to study and use the molecular descriptors (Dragon 7.0) as independent variables. In the mathematical simulation approach, hardness (N), water activity and adhesiveness (mJ) were assumed as the dependent variables.

## 2. Materials and Methods

### 2.1. Materials

For processing into jam, blueberries of the Climax Rabbiteye (most commonly planted Rabbiteye blueberry cultivar in Brazil, whose bush has green elliptical foliage that produces large light blue fruits) and Powder Blue Rabbiteye (native to the warm regions of the southern U.S., medium size with a very light blue color, small dry scars, and average firmness and flavor) varieties were pulped by pulper. Conventional products were processed according to Santos et al. [38] and Guimarães et al. [24], using the proportion pulp/sugar of 3/2 with HM pectin added at a ratio of 1.0% of pulp quantity. The pulp, added to 30% of the sucrose amount, was processed in a stainless steel-jacketed cooker until the mixture reached 35° Brix (measured in refractometer). Once the consistency was reached, the remaining sugar with hydrated pectin (in 0.15 kg of water for each 0.008 kg of pectin) was added and the mixture was kept heated until it reached 67° Brix. Synthetic HM pectin and HM pectin extracted from the passion fruit peel were used for the Powder Blue and Climax fruit varieties, respectively, since these formulations were the most accepted, according to Guimarães et al. [24].

For the extraction of pectin from passion fruit peel, the same methodology followed by Guimarães et al. [24] was applied, where a mixture of passion fruit peel and water, in a 1:1 ratio, was well homogenized, with the pH adjusted to 3.0 by adding 3% citric acid. Then, the mixture was heated to a boiling point for 30 min and, after cooling, it was filtered through a thin cloth and 95% ethanol was added until pectin was precipitated. Such processing resulted in the extraction of pectin, with a final moisture content of 94.08%.

The preparation of the light formulations followed Granada et al. [8] and Lago et al. [39] using 50% of the sucrose amount used for conventional jellies and LM pectin (on 1.5% pulp amount) was added at once in a stainless steel-jacketed cooker until the product reached a total solid content corresponding to 37° Brix. In this stage, CaCl_2_ was added (55 mg per gram of pectin proportion). A mix of xantana and carrageenan gums (1:1 *w*/*w*) was used, on 2% of sucrose weight proportion, to increase the light jam consistency and coloration.

After processing, the jelly was hot filled in 250 g glass containers, closed with a metal lid (previously sanitized), inverted for 15 min, and then cooled with cold water and stored at room temperature.

### 2.2. Analytical Methods

The influence of storage on the characteristics of the jams was verified through the following physicochemical analyses: pH ([40] Method 981.12), acidity ([40] Method 942.15); soluble solids ([40] Method 932.12), nonreducing and reducing sugars [41]; anthocyanins [42], water activity (in Alpax 650 AW meter) and textural properties at the following times: immediately after processing (zero time) and after 30, 90 and 120 days.

For the quantification of anthocyanins, 10 g of the sample were homogenized with an extracting solution (95% ethanol: 1.5 M HCl—85:15 *v*/*v*) and stored for 12 h at 4 °C. After 12 h, the samples were filtered, and the residues washed using the extractor solution until the complete removal of the pigments. The filtrates were then collected in 100 mL volumetric flasks, checked with the extracting solution, and left to rest in the absence of light at room temperature, for two hours, when the absorbance at 532 nm was measured. Anthocyanin content was estimated as cyanidin-3-gucoside at 532 nm, using a molar absorptivity coefficient of 26,900 L/cm.mol and molecular weight of 449.2 g/mol [40].

The rheological measurements were performed using a texturometer (Texture Analyzer, TA-TX2, manufactured by Brookfiels Engineering Laboratories, INC, Middleboro, Massachusetts, USA) with a 1.0 cm^2^ probe (TA3/100), 30 mm and pretest, test and posttest velocities equal to 2, 1 and 1 mm/s, respectively. The texturometer has a load capacity of 4500 g, and is widely used for measuring parameters related to the consistency and/or texture of the gels. The principle of operation of the texturometer is based on the application of a load on a sample and this load is applied with adjustable penetration speeds, being able to operate both for penetration and for traction, through different types of probes, which are chosen according to the material to be tested and the consistency and/or texture parameter to be analyzed. The probe used for measurements in gels is cylindrical, with a diameter of 38 mm, where the test consisted of a penetration of 30.0 mm into the product, and the necessary force was verified. The results, obtained from the force × time curve, were calculated by the Texture Expert Software Version 1.22.

The analyzed parameters were hardness (which is related to the physical force, in N or Newton, of the first bite, that is, to the maximum force applied in the first compression cycle of the sample) and adhesion (which corresponds to a negative force (in Newton) resulting from the work required to overcome the attraction between the sample and the probe; that is, it is a surface characteristic that depends on the combination of adhesion forces and cohesion). These analyses of the rheological properties (related to the texture of the product) were performed in triplicate, where the average of the replicates served as data for statistical analyses and artificial neural network simulation. Both hardness and adhesiveness have been constantly determined in scientific works to characterize the rheological behavior of jellies [43,44,45,46].

### 2.3. Statistical Analysis

The analysis of the results referring to the physicochemical and rheological properties of the product followed the procedure described by Guimarães [16], according to the following statistical methodologies: (i) Analysis of Variance (ANOVA), which, according to Ferreira [47], measures the level of significance of the main effects and interactions; and (ii) Regression analysis, which evaluates the influence of time on changes during storage. According to ANOVA, the results had no significant effect and were not submitted to the regression analysis.

### 2.4. Simulation with Artificial Neural Networks

The physical, chemical and rheological properties of blueberry jam were analyzed to study the capabilities of artificial neural networks (ANNs) to learn, test and predict the properties: hardness (N), water activity and adhesiveness (mJ). The main learning variables for the ANN method developed in this research were the following properties: time (days), % water, % citric acid, % glucose, % sucrose, % anthocyanin, % HM pectin, % LM pectin, % xanthan gum, pH and the acidity (%), all considered as independent variables to define the physical, chemical and rheological properties of four types of blueberry jam. To distinguish between the physical and rheological characteristics of the blueberry jams studied in this work, molecular descriptors were obtained from computational chemistry (Dragon 7), which are shown in Table 1.

In this work, four types of blueberry jams were studied, identified as: FA, FB, FC and FD. In each blueberry jam, the % water, % citric acid, % glucose, % sucrose or % anthocyanin is the highest value (in mass percentage). The effect of time on the physical and chemical properties, based on the ANOVA summary, is shown for the four products, where FA is jam with synthetic high methoxyl pectin (HM) and Powder Blue fruit variety; FB is light jam with Powder Blue fruit variety and synthetic low methoxyl amidated (LM) pectin; FC is jam with high methoxyl pectin (HM) extracted from passion fruit and Climax fruit variety; and FD is light jam with Climax fruit variety and synthetic low methoxyl amidated (LM) pectin. To this end, these variables were used to determine the molecular descriptors according to the mass percentage. First, by using the water, citric acid, glucose, sucrose and anthocyanin molecules, the SMILES code for each component was found. Second, based on the SMILES codes, the numeric values for the fifteen molecular descriptors were obtained using Dragon 7 (Table 1).

Representative molecular descriptors of the jam were obtained using the mass percentages of each component for a given time and for each type of blueberry jam using the following simple equation:

(1) $${MD}_{BLUEBERRY\;JAM}=\sum_{1}^{5}{\;\%}_{COMPONENT}*{MD}_{COMPONENT}$$

where *%_COMPONENT_* and *MD_COMPONENT_* represent the mass percentage and the molecular descriptor of the component (water, citric acid, glucose, sucrose, and anthocyanin). Molecular descriptors of the blueberry jam were considered as independent variables of % HM pectin, % LM pectin, % xanthan gum, pH and acidity (%). Dependent variables used in this work for the simulation of the physical, chemical and rheological properties of blueberry jam were hardness (N), water activity and adhesiveness (mJ).

The methodology used for the learning, testing and prediction of the physical, chemical and rheological properties of the four types of blueberry jams was the same used in other previous works [34,35]. In this work, a spreadsheet file (MS-Excel) was also used with six worksheets. The first, third and fifth worksheets for the learning, testing and prediction steps, respectively, contain the independent variables. The second, fourth and sixth worksheets for the learning, testing and prediction steps, respectively, contain the dependent variables. The data and amount of data were different for the first, third and fifth worksheets. A computer routine, developed in MatLab [48], was adapted for the three dependent variables [31]. The routine interacts with each worksheet of the spreadsheet file. More information about these interactions between the spreadsheet and the routine for each step can be found in our previous works.

## 3. Results and Discussion

### 3.1. Experimental

Table 2 shows the calculated F values, where the comparison of the means of these variables at different times was performed using the ANOVA, where the physicochemical properties highlighted with * are those that showed significant interaction with time (i.e., where the F_cal_ value resulted in a value greater than Ftab).

The F_calc_ value is obtained by dividing the mean square of the factor (obtained by dividing the sum of squares by the degrees of freedom) by the mean square of the error. For the determination of F_tab_, consult the F distribution table of Snedecor F_x,y,5%_, where x is the degrees of freedom of the factor, y is the degrees of freedom of the error and the significance or the error of the type α is 5% (probability of the accepted hypothesis being false).

In other words, Table 2 outlines the influence of time on the physicochemical properties of the jellies, based on the ANOVA summary, where the Fcalc values were obtained from the means of the three repetitions, referring to the physical and chemical analyses of the four different formulations of jam (Supplementary Material: Tables S1 and S2).

Based on the results in Table 2, the regression models at the 5% significance level were obtained and are shown in Table 3, where x corresponds to the independent variable (time) and y is the response variable (such as reducing sugars, anthocyanins, soluble solids or water activity). The adequacy of such models can be examined by the coefficient of determination (*R*^2^). According to the results presented in Table 3, it is verified that the linear model did not fit properly with the experimental data relating time with the soluble solids content in the traditional jelly made with fruits of the Climax variety and HM pectin extracted from the peel of the passion fruit (FC).

According to Table 2, it is noted that in all products, there was no significant interaction of pH and acidity with time, and, in the case of light products, they also did not show significant interaction between time and content of soluble solids. However, for conventional products (FA and FC), it is possible to notice a significant interaction of this property with time, which points to a possible tendency for the product to crystallize during storage [49]. In the present work, the use of HM synthetic pectin (in the FA) favored the crystallization of the jelly compared with the pectin extracted from the passion fruit peel (in the CF) since, in the latter, the level of hydration is very high. The water activity varied significantly over time only the product prepared with the Climax variety and pectin extracted from passion fruit (FC), which can be explained by the occurrence of sucrose hydrolysis, which was confirmed by the increase in the reducing sugar content.

Regarding the reduction in anthocyanin content during storage, the results presented in Table 2 show that such reduction was not significant, except for the conventional product made with synthetic HM pectin (FA). According to Guimarães et al. [24], under these conditions, the anthocyanin content was lower in the jelly immediately after processing (i.e., at zero storage time) in relation to the other formulations (FB, FC and FD), due to the longer cooking time and the higher concentration of sucrose. Thus, the lower proportion of anthocyanins in the FA inhibited possible interactions of the pigment with ascorbic acid, oxygen or light during storage. In contrast, Howard et al. [23], when analyzing the effect of storage time on the polyphenolic compounds of blueberry jam, found that sugar-free products retained higher levels of anthocyanins than traditional jams at the end of storage. However, in the present work, the light products are not free of sugar since they were prepared with 50% of the amount of sugar in conventional jellies and, therefore, this proportion of sugar was sufficient to reduce the stability of the anthocyanins due to the production of polymers from degradation products. Additionally, the results of the anthocyanin content of the jellies produced in the present work corroborate the experimental results achieved by Melgarejo et al. [50], who, when analyzing the influence of storage time on pomegranate jelly, found great instability of these phenolic compounds with time for the product stored at room temperature. Sellappan et al. [51] classified blueberries and blackberries as rich sources of anthocyanins and, although cooking can reduce the content of these pigments, jam can still be considered a source of this phenolic compound.

Table 4 shows the influence of time on the rheological parameters and, from these, the regression models (at a level of 5% of significance) were set, according to Table 5.

The results presented in Table 4 show that, in all products, time significantly influenced adhesiveness, while hardness was not significantly influenced by time when the product was prepared with the Climax fruit variety and pectin extracted from passion fruit peel. (i.e., in the FC formulation, the gel remained stable). Regarding hardness in the other formulations (FA, FB and FD), the negative angular coefficient present in the regression equations shows a reduction of this property over storage time, possibly due to syneresis. The same effect was observed by Dias et al. [52] when analyzing the effects of temperature on changes in the physicochemical and microbiological properties of jam made from the banana (*Musa* spp.) peel.

The adhesiveness of the traditional jam prepared from the Powder Blue fruit (FA) increased significantly during storage, most likely due to the increase in the soluble solids content, with a consequent decreasing in the moisture content [53]. In contrast, since the pectin extracted from the passion fruit peel is highly hydrated, it caused the adhesiveness of the traditional jelly prepared from the Climax fruit (FC) to reduce over time during storage, which is consistent with Oliveira et al. [11], who, when analyzing the stability of traditional umbu-caja jam during storage, reported a tendency for adhesiveness to reduce during storage. For light products, FB and FD presented similar fluidity, showing no effect of the fruit variety on the texture properties of the jams.

### 3.2. Prediction

#### 3.2.1. SMILES Code

The SMILES codes generated for the main components present in the blueberry jam, are shown in Table 6.

#### 3.2.2. Molecular Descriptor

Numeric values of the selected molecular descriptors, obtained by using the SMILES codes (Table 6) and the Dragon 7.0 software, are shown in Table 7.

#### 3.2.3. Artificial Neural Networks

To find the most accurate artificial neural network, several network architectures composed of three and four layers, with different numbers of neurons in each layer, were tested. The optimum number of layers and neurons was determined by trial and error because there is no information about the optimum number of layers and neurons for the calculation of properties for any type of blueberry jam. Furthermore, specific conditions were imposed, because the architecture had to be simple and results have to have the best accuracy [27].

**Figure 2:**
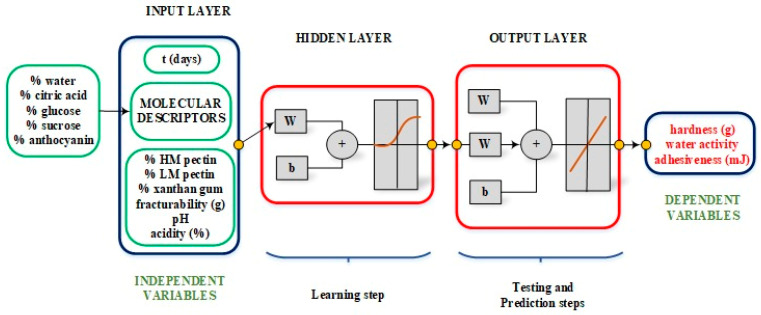
Architecture for the ANN model used in this work.

Generally, the ANN analysis is made in three basic steps: learning, testing, and prediction (Figure 2) [29]. In this work, 585 points were used for the learning step, 55 points were used in testing and 40 points were set aside for the prediction step. In all steps, the information of each point was different. This was achieved using “random separation” of the data available in these three groups. It is very important if users want to have a network with good and acceptable predicting capabilities. Several architectures were used and run to find the most appropriate for this study. The calculation methodology used was as follows: first, the configuration with three layers was used and, second, the configuration with four layers, considering that each layer was composed of different nodes.

In this manuscript, the criteria in the ANNs of previous research works were maintained [32,54]: feed forw4rd back propagation, the error between iterations was set to 0.0001, the maximum number of iterations was set to 500 and the computer program was developed to run 50 times (Levenberg–Marquardt function). Table 8 shows some characteristics of the AAN model.

The average absolute deviation and the maximum absolute deviation were evaluated in function of the appropriate selection of data and the best selection of the architecture and variables. From this choice, an acceptable correlation between the data and the predicted properties was found in terms of the physical, chemical, and rheological properties of blueberry jams and of new cases not used for learning or testing.

The optimized architecture for the artificial neural network was composed of four layers (10-30-10-3): an input layer with 10 neurons, two hidden layers with 30 and 10 neurons, and an output layer with three neurons. A sigmoid hyperbolic tangent activation function was used for the two hidden layers, and a linear activation function was used for the output layer.

The weight matrices and diagonal vectors for the optimized ANN model are listed in the Supplementary Material (Tables S3–S5) for the interaction between the input layer and the hidden layer, the first hidden layer and the second hidden layer, and the second hidden layer and the output layer, respectively. In these tables, w^I^ represents the weight matrix (thirty lines × ten columns) for the connection between the input layer and the first hidden layer, w^H1^−H2 is the weight matrix (ten lines × thirty columns) for the connection between the first and the second hidden layer, w^H2^ represents the weight matrix (three lines × ten columns) for the connection between the second hidden layer and the output layer, b^H1^ is the diagonal vector (thirty lines) of the first hidden neuron, b^H1^−H2 is the diagonal vector (ten lines) of the second hidden neuron, and, finally, b^0^ represents the diagonal vector (three lines) of the output neurons. The accuracy of this optimized configuration was checked for the prediction step. Results, in terms of the lowest maximum absolute deviations for the three dependent variables, confirm that the configuration for the run 44 is the best (Supplementary Material: Table S10).

The results, in terms of the maximum absolute deviations for the other analyzed architectures, such as X-X-3, 3-X-X-3, 5-X-X-3, 7-X-X-3, 10-X-X-3 and 15-X-X-3, are shown in the Supplementary Material (Tables S6–S11). In these tables, the average absolute deviations |%Δ hardness (N)|, |%Δ water activity| and |%Δ adhesiveness (mJ)|, for a set of N data, are defined as:

(2) $$\left|\%∆\;DV\right|=\frac{100}{N}\sum_{1}^{N}\frac{{\left|{\left(DV\right)}^{pred}-{\left(DV\right)}^{exp}\right|}_{i}}{{\left(DV\right)}_{i}^{pred}}$$

where *DV* represents any of the dependent variables: hardness (N), water activity or adhesiveness (mJ).

In the learning step, individual absolute deviations between correlated and experimental values of the physical, chemical and rheological properties of blueberry jam were below 5.0% for most of the data. For the hardness (N), 105 points showed absolute deviations greater than 2.0%, with 7.85% being the highest value. For water activity, 90 points showed absolute deviations greater than 3.0% (4.78% for the highest value), and for adhesiveness (mJ), 120 points showed absolute deviations greater than 2.6% (3.85% for the highest value), while all the other 270 points gave absolute deviations below 1.0%. The average absolute deviations for the hardness (N), water activity and adhesiveness (mJ) are 1.8%, 1.5% and 2.1%, respectively. These values indicate that the ANN learned correctly.

Once the learning step was successfully performed and the optimal network architecture was determined, 55 and 40 input data points (independent variables) of the four types of blueberry jams, not used in the learning process, were included in the third (testing step) and fifth (prediction step) worksheets of the excel file, respectively, and were read by the computer program. For the testing (Table 9) and the prediction step (Table 10), some of the 30 and 19 values tested for the three dependent variables studied are shown.

Table 9 shows the average individual absolute deviations for the hardness (N), water activity and adhesiveness (mJ), tested by the proposed ANN architecture for some selected points not used in the learning process. In most cases, results for the ANN configuration show the hardness (N) with absolute deviations below 0.70%, water activity with absolute deviations below 0.39%, and adhesiveness (mJ) with absolute deviations below 1.48%, except for two cases where absolute deviations were below 4.63%.

The average absolute deviations for hardness (N), water activity and adhesiveness (mJ), predicted by the best ANN architecture are shown in Table 10. Data used in this process are different than those used during the learning and testing steps. In Table 10, the best ANN model is capable of reproducing the hardness (N), water activity and adhesiveness (mJ) of the four types of blueberry jams with average absolute deviations between 0.06%, 0.07% and 0.13%, respectively, being that the maximum individual absolute deviation in predicting the hardness (N) is 0.43%. The water activity is predicted, in all cases studied, with average absolute deviations from 0.00% to 0.39%, and for the adhesiveness (mJ), the average absolute deviations vary from 0.00% to 0.43%. In the Supplementary Material, results obtained for the prediction step of the hardness (N), water activity and adhesiveness (mJ) are plotted for the six architectures studied (Figures S1–S6). It is important to note from these figures that only the architecture 10-X-X-3 presents the minimum deviations (less than 0.4%) for the three dependent variables, when compared to the other architectures. Another comparison that can be made is through the average of the deviations of the three dependent variables.

If the optimal configurations of the six architectures studied are compared (10-10-3, 3-2-20-3, 5-25-30-3, 7-10-20-3, 10-30-10-3 and 15-25-10-3) (Supplementary Material: Figure S7), the minimum average deviation is minimum for the configuration 10-30-10-3. It can also be seen that with the three-layer architecture, the average deviation is the highest of all the architectures.

## 4. Conclusions

This study demonstrated that longer cooking times and higher amounts of sucrose in traditional formulations, despite resulting in jellies with less anthocyanin content (compared to light products), can contribute to the inhibition of possible interactions of pigment (with oxygen, light or ascorbic acid). The inhibition of these interactions resulted in lower losses of this phenolic compound over the storage period.

The traditional jelly made from Climax fruits and HM pectin extracted from passion fruit peel (FC) was the most influenced by storage time with respect to physical and chemical properties. In this case, in addition to the reduction in the anthocyanin content, the storage time significantly influenced the content of reduced sugars and soluble solids, as well as water activity.

Regarding the rheological properties, with the exception of the hardness of the FC product (made from Climax fruits variety and HM pectin extracted from the passion fruit peel), practically all the texture parameters were influenced by storage time. In this context, the conventional product prepared with fruits of the Powder Blue variety and synthetic HM pectin (FA) was the most affected, in terms of hardness and adhesiveness of the final product.

The variables of time (days), % water, % citric acid, % glucose, % sucrose, % anthocyanin, % pectin HM, % Pectin LM, % xanthan gum, pH and acidity (%) were used as independent variables in an artificial neural network model to predict the physical, chemical and rheological properties of four types of blueberry jam. Mathematical simulations, using different architectures of an ANN model, were also implemented using molecular descriptors as independent variables to predict the physical, chemical and rheological properties of blueberry jam. Experimental data composed of 680 points were used (independent and dependent variables), 585 points were used in the learning step, 55 points were used in testing and 40 points were set aside for the prediction step. ANN architectures were developed with three and four layers, and the four-layer architecture was found to be the most accurate, represented as 10-30-10-3 (10 is the number of nodes or independent variables in the input layer, 30 and 10 are the numbers of nodes in the hidden layers, and 3 is the number of nodes or dependent variables in the output layer). Numerical results were obtained in function of general mean absolute deviations, which were less than 0.06%, 0.07% and 0.13% for each dependent variable: hardness (N), water activity and adhesiveness (mJ).

## Figures and Tables

**Table 1:** Molecular descriptors from Dragon 7.0 used.

Molecular Descriptors	Name	Subblock	Block
MW	Molecular weight	Basic descriptors	Constitutional indexes
AMW	Average molecular weight		
nH	Number of Hydrogen atoms		
nC	Number of carbon atoms		
nStructures	Number of disconnected structures		
Pol	Polarity number	Distance-based indexes	Topological indexes
X1Av	Average valence connectivity index of order 1	Kier–Hall molecular connectivity indexes	Connectivity indexes
X1SOL	Solvation connectivity index	Solvation connectivity indexes	
XMOD	Modified Randic index	Randic-like connectivity indexes	
RDCHI	Reciprocal distance sum Randic-like index		
P_VSA_p_1	P_VSA-like polarizability, bin 1	Polarizability	P_VSA_like descriptors
nHDon	Number of donor atoms for H-bonds	Basic descriptors	Functional group counts
SAtot	Surface area (total)	Basic descriptors	Molecular properties
VvdwMG	Van der Waals volume for McGowan volume		
PDI	Packing density index		

**Table 2:** Summary of the ANOVA referring to the physicochemical properties of blueberry jam during storage.

Product	F_cal_							
	F_tab_	pH	Acidy	Nonreducing Sugars (Sucrose)	Reducing Sugars (Glucose)	Anthocyanins	Soluble Solids	Water Activity
FA	3.240	0.034	2.390	1.347	0.0904	1.165	16.468 *	2.286
FB	3.240	0.080	0.065	1.759	0.325	23.439 *	1.429	0.096
FC	3.240	0.222	2.286	0.214	5.912 *	53.650 *	3.516 *	11.636 *
FD	3.240	0.584	1.913	1.755	1.040	41.514 *	0.169	0.973

* are the physicochemical properties that showed significant interaction with time.

**Table 3:** Linear regression equations for the physicochemical analyzes of blueberry jam.

Variable Response	Product	Estimating Model	*R* ^2^
Reducing sugars	FC	y=64.79−0.083x	0.75
Anthocyanins	FB	y=53.09−0.117x	0.92
Anthocyanins	FC	y=39.25−0.136x	0.96
Anthocyanins	FD	y=73.52−0.251x	0.95
Soluble solids	FA	y=47.85+0.046x	0.89
Soluble solids	FC	y=52.11−0.009x	0.08
Water activity	FC	y=0.73−0.001x	0.85

**Table 4:** Summary of ANOVA of the rheological properties of blueberry jam during storage.

Product	F_cal_		
	F_tab_	Hardness	Adhesiveness
FA	3.240	13.083 *	95.474 *
FB	3.240	24.147 *	79.775 *
FC	3.240	0.199	7.234 *
FD	3.240	17.982 *	5.272 *

* are the physicochemical properties that showed significant interaction with time.

**Table 5:** Linear regression equations for the rheological (texture) parameters of blueberry jam.

Variable Response	Product	Estimating Model	*R* ^2^
Hardness	FA	y=11.64−0.027x	0.87
Hardness	FB	y=6.37−0.018x	0.92
Hardness	FD	y=6.01−0.011x	0.90
Adhesiveness	FA	y=86.29+0.583x	0.78
Adhesiveness	FB	y=34.42−0.146x	0.96
Adhesiveness	FC	y=68.41−0.260x	0.78
Adhesiveness	FD	y=33.05−0.104x	0.56

**Table 6:** SMILES code for the main components contained in the blueberry jam.

Component	SMILES Code
Water	O
Citric acid	OC(=O)CC(O)(CC(O)=O)C(O)=O
Glucose	OC[C@@H](O)[C@@H](O)[C@H](O)[C@@H](O)C=O
Sucrose	OC[C@H]1O[C@H](O[C@]2(CO)O[C@H](CO)[C@@H](O)[C@@H]2O)[C@H](O)[C@@H](O)[C@@H]1O
Anthocyanin	C1CCC(CC1)C2CCC3CCCCC3[O+]2

**Table 7:** Numeric values for the molecular descriptors (MD) used in this work.

Molecular Descriptor	Water	Citric Acid	Glucose	Sucrose	Anthocyanin
MW	18.020	192.140	180.180	342.340	207.260
AMW	6.007	9.150	7.507	7.608	7.676
nH	2	8	12	22	11
nC	0	6	6	12	15
nStructures	1	1	1	1	1
Pol	0	16	16	43	23
X1Av	0	0.264	0.302	0.294	0.297
X1sol	0.000	5.776	5.540	10.807	7.933
XMOD	0.000	38.622	36.964	72.190	48.412
RDCHI	0.000	2.190	2.154	3.143	2.966
P_VSA_p_1	0.000	47.084	82.398	164.795	129.482
nHDon	2	4	5	8	0
SAtot	58.196	301.051	332.384	539.621	259.272
VvdwMG	14.767	87.516	89.375	155.050	114.516
PDI	0.478	0.682	0.631	0.686	1.046

**Table 8:** Some characteristics of the ANN model.

Type of Network	Training Algorithm
Feed-Forward Backpropagation (newff MatLab function)	Levenberg–Marquardt backpropagation (trainln MatLab function)

**Table 9:** Some average individual deviations for the hardness (N), water activity and adhesiveness (mJ) for the testing step of the configuration 10-30-10-3.

Blueberry Jam	Experimental						Experimental			Testing with ANN			% Deviation		
	Time	HM	LM	xf	pH	ac	Hard	wa	adhe	Hard	wa	adhe	Hard	wa	adhe
FB	10	0.0	1.2	2	3.77	0.39	6.25	0.90	66.55	6.26	0.90	66.50	0.06	0.02	0.08
FD	78	0.0	1.2	2	3.49	0.44	5.04	0.90	25.07	5.04	0.90	25.13	0.09	0.06	0.24
FB	28	0.0	1.2	2	3.98	0.34	5.92	0.85	60.38	5.93	0.85	60.68	0.27	0.00	0.50
FB	34	0.0	1.2	2	3.99	0.34	5.78	0.85	58.59	5.80	0.85	58.57	0.29	0.03	0.03
FA	92	0.6	0.0	0	3.42	0.57	9.26	0.75	27.57	9.27	0.75	28.91	0.07	0.19	4.63
FB	30	0.0	1.2	2	3.97	0.34	5.88	0.84	59.75	5.92	0.84	60.09	0.70	0.04	0.57
FC	66	0.6	0.0	0	3.25	0.69	6.64	0.78	22.14	6.65	0.78	22.07	0.12	0.01	0.34
FD	84	0.0	1.2	2	3.48	0.44	4.93	0.89	25.34	4.94	0.89	25.32	0.17	0.10	0.08
FA	98	0.6	0.0	0	3.44	0.58	9.02	0.77	25.86	9.02	0.77	26.25	0.13	0.02	1.48
FD	24	0.0	1.2	2	3.51	0.43	5.90	0.91	25.89	5.89	0.91	25.88	0.04	0.05	0.05
FB	62	0.0	1.2	2	3.84	0.38	5.08	0.89	50.45	5.08	0.89	50.52	0.01	0.03	0.13
FA	62	0.6	0.0	0	3.37	0.56	10.38	0.75	49.41	10.38	0.75	49.47	0.02	0.00	0.13
FA	22	0.6	0.0	0	3.32	0.56	11.43	0.75	77.78	11.44	0.75	77.81	0.17	0.05	0.05
FD	92	0.0	1.2	2	3.47	0.44	4.83	0.89	25.30	4.82	0.89	25.30	0.25	0.39	0.03
FC	72	0.6	0.0	0	3.25	0.69	6.74	0.78	21.75	6.75	0.78	21.67	0.04	0.00	0.34
FB	54	0.0	1.2	2	3.91	0.37	5.28	0.88	52.78	5.28	0.88	52.90	0.04	0.02	0.23
FD	112	0.0	1.2	2	3.49	0.40	4.85	0.92	22.13	4.85	0.92	22.11	0.10	0.05	0.10
FD	14	0.0	1.2	2	3.42	0.44	5.91	0.90	30.87	5.90	0.90	30.93	0.02	0.06	0.19
FB	48	0.0	1.2	2	3.95	0.36	5.43	0.87	54.52	5.43	0.87	54.50	0.04	0.01	0.04
FA	94	0.6	0.0	0	3.43	0.57	9.18	0.76	27.00	9.19	0.76	27.97	0.12	0.11	3.48
FB	80	0.0	1.2	2	3.69	0.40	4.63	0.92	45.23	4.61	0.92	44.94	0.26	0.08	0.63

Independent variables: time (days); HM: % HM pectin; LM: % LM pectin; xf: % xanthan gum; pH; ac: acidity (%). Dependent variables: hard: hardness (N); wa: water activity; adhe: adhesiveness (mJ).

**Table 10:** Some average individual deviations for the hardness (N), water activity and adhesiveness (mJ) for the prediction step of the configuration 10-30-10-3.

Blueberry Jam	Experimental						Experimental			Predicted with ANN			% Deviation		
	Time	HM	LM	xf	pH	ac	Hard	wa	adhe	Hard	wa	adhe	Hard	wa	adhe
FB	22	0.0	1.2	2	3.94	0.36	6.03	0.86	62.48	6.03	0.86	62.53	0.09	0.01	0.09
FD	106	0.0	1.2	2	3.48	0.41	4.84	0.91	23.08	4.84	0.91	23.10	0.05	0.07	0.08
FB	120	0.0	1.2	2	3.63	0.36	4.51	0.91	39.54	4.53	0.90	39.51	0.43	0.77	0.07
FB	78	0.0	1.2	2	3.70	0.40	4.67	0.91	45.81	4.68	0.91	45.68	0.02	0.04	0.28
FB	110	0.0	1.2	2	3.65	0.38	4.47	0.91	40.46	4.47	0.91	40.48	0.05	0.03	0.05
FD	16	0.0	1.2	2	3.44	0.44	5.90	0.90	29.87	5.90	0.90	29.86	0.05	0.01	0.05
FB	42	0.0	1.2	2	3.98	0.35	5.58	0.86	56.26	5.58	0.86	56.24	0.01	0.04	0.04
FB	40	0.0	1.2	2	3.99	0.35	5.63	0.86	56.85	5.64	0.85	56.82	0.04	0.05	0.04
FD	86	0.0	1.2	2	3.47	0.44	4.90	0.89	25.43	4.90	0.89	25.43	0.05	0.06	0.02
FC	6	0.6	0.0	0	3.24	0.69	6.77	0.73	35.89	6.80	0.73	35.87	0.07	0.03	0.06
FD	70	0.0	1.2	2	3.50	0.44	5.18	0.90	24.71	5.17	0.90	24.82	0.06	0.03	0.43
FB	68	0.0	1.2	2	3.79	0.39	4.93	0.90	48.71	4.93	0.90	48.77	0.05	0.02	0.12
FD	104	0.0	1.2	2	3.48	0.41	4.84	0.91	23.40	4.83	0.90	23.40	0.09	0.06	0.02
FC	106	0.6	0.0	0	3.26	0.70	6.96	0.79	19.60	6.96	0.79	19.55	0.00	0.01	0.26
FA	54	0.6	0.0	0	3.37	0.56	10.68	0.75	55.63	10.68	0.75	55.58	0.00	0.01	0.09
FC	80	0.6	0.0	0	3.24	0.69	6.88	0.79	21.22	6.88	0.79	21.24	0.00	0.00	0.07
FD	66	0.0	1.2	2	3.51	0.44	5.26	0.90	24.53	5.26	0.90	24.60	0.06	0.02	0.30
FB	72	0.0	1.2	2	3.75	0.39	4.83	0.90	47.55	4.83	0.90	47.55	0.02	0.04	0.00
FA	100	0.6	0.0	0	3.45	0.58	8.93	0.77	25.29	8.93	0.77	25.40	0.04	0.02	0.42

Independent variables: time (days); HM: % HM pectin; LM: % LM pectin; xf: % xanthan gum; pH; ac: acidity (%). Dependent variables: hard: hardness (N); wa: water activity; adhe: adhesiveness (mJ).

## Data Availability

Data sets generated during the current study are available from the corresponding author on reasonable request.

## Supplementary Materials

The following supporting information can be downloaded at: https://www.mdpi.com/article/10.3390/foods12152853/s1, Table S1: Means and standard deviations of the results of the physicochemical analyses of the blueberry jams of the Climax and Powder Blue varieties during the storage period. Table S2: Texture analysis results of Climax and Powder blue blueberry jellies. Table S3: Weights and bias of the optimized ANN architecture for the Input—First Hidden layer connections. Table S4: Weights and bias of the optimized ANN architecture for the First Hidden layer—Second Hidden layer connections. Table S5: Weights and bias of the optimized ANN architecture for the Second Hidden layer—Output connections. Table S6: Results obtained for the three dependent variables for the prediction step for architectures X-X-3. Table S7: Results obtained for the three dependent variables for the prediction step for architectures 3-X-X-3. Table S8: Results obtained for the three dependent variables for the prediction step for architectures 5-X-X-3. Table S9: Results obtained for the three dependent variables for the prediction step for architectures 7-X-X-3. Table S10: Results obtained for the three dependent variables for the prediction step for architectures 10-X-X-3. Table S11: Results obtained for the three dependent variables for the prediction step for architectures 15-X-X-3. Figure S1: Architecture X-X-3. Figure S2: Architecture 3-X-X-3. Figure S3: Architecture 5-X-X-3. Figure S4: Architecture 7-X-X-3. Figure S5: Architecture 10-X-X-3. Figure S6: Architecture 15-X-X-3. Figure S7: Optimal architectures for three and four layers.
