# Supplementary material for: Effect of Storage Time on the Physical, Chemical, and Rheological Properties of Blueberry Jam: Experimental Measurements and Artificial Neural Network Simulation
Source: Foods. 2023 Jul 27;12(15):2853. doi: 10.3390/foods12152853 (PMC10418431; doi:10.3390/foods12152853)
Supplement: Supplementary file 1 [file foods-12-02853-s001.zip › foods-2472237-supplementary.pdf]

foods

Supplementary Materials

Article

Effect of Storage Time on the Physical, Chemical, and Rheological Properties of Blueberry Jam: Experimental Measurements and Artificial Neural Network Simulation

Daniela Helena Pelegrine Guimarães [1], Ana Lúcia Gabas Ferreira [2,*] and Pedro Felipe Arce[1]

[1] Department of Chemical Engineering, Engineering School of Lorena, University of São Paulo, Lorena 12602-810, SP, Brazil; dhguima@usp.br (D.H.P.G.); parce@usp.br (P.F.A.)
[2] Department of Basic and Environmental Sciences, Engineering School of Lorena, University of São Paulo, Lorena 12602-810, SP, Brazil
* Correspondence: gabas@usp.br

Table S1. Means and standard deviations of the results of the physicochemical analyses of the blueberry jams of the Climax and Powder Blue varieties during the storage period.

	Storage (Days)	pH	Acidy (%)	Anthocyanin (mg/100 g)	Reducing Sugar (%)	Water Activity	Soluble Solids (°Brix)
FA	0	3.23 ± 0.057 [a]	0.57 ± 0.067 [a]	29.57±5.08 [a]	50.49±1.75 [a]	0.75±0.01 [a]	48.7±0.001 [a]
	30 days	3.32 ±0.046 [a]	0.56±0.029 [a]	31.85 ± 4.63 [a]	52.77 ± 1.25 [a]	0.75 ± 0.11 [a]	48.0 ± 0.001 [a]
	90 days	3.23 ± 0.056 [a]	0.57 ± 0.067 [a]	29.57 ± 1.80 [a]	54.96 ± 3.95 [a]	0.75 ± 0.06 [a]	52.3 ± 0.002 [b]
	120 days	3.26 ± 0.015 [a]	0.60 ± 0.028 [a]	28.19 ± 3.78 [a]	47.60 ± 1.39 [a]	0.82 ± 0.01 [a]	53.5 ± 0.002 [b]
	MSD						3.32
FB	0	3.52 ± 0.040 [a]	0.42 ± 0.016 [a]	54.95 ± 4.05 [a]	32.87 ± 0.72 [a]	0.928 ± 0.01 [a]	35.1 ± 0.001 [a]
	30 days	3.97 ± 0.030 [a]	0.34 ± 0.064 [a]	46.97 ± 3.09 [b]	35.89 ± 0.18 [a]	0.836 ± 0.02 [a]	35.0 ± 0.001 [a]
	90 days	3.52 ± 0.040 [a]	0.42 ± 0.016 [a]	42.92 ± 1.51 [b]	34.80 ± 5.33 [a]	0.928 ± 0.01 [a]	35.1 ± 0.003 [a]
	120 days	3.63 ± 0.041 [a]	0.36 ± 0.077 [a]	39.41 ± 1.57 [b]	34.39 ± 1.86 [a]	0.907 ± 0.02 [a]	34.8 ± 0.001 [a]
	MSD			7.25			
FC	0	3.23 ± 0.012 [a]	0.69 ± 0.088 [a]	39.96 ± 2.5 [a]	63.58 ± 2.47 [a]	0.719 ± 0.01 [a]	53.3 ± 0.001 [a]
	30 days	3.28 ± 0.068 [a]	0.69 ± 0.006 [a]	33.67 ± 3.30 [b]	62.75 ± 1.49 [a]	0.748 ± 0.04 [a]	50.3 ± 0.002 [b]
	90 days	3.23 ± 0.012 [a]	0.69 ± 0.009 [a]	28.65 ± 3.2 [c]	60.77 ± 5.71 [a]	0.788 ± 0.05 [b]	54.3 ± 0.002 [ca]
	120 days	3.28 ± 0.014 [a]	0.71 ± 0.021 [a]	22.05 ± 4.50 [d]	52.09 ± 1.78 [b]	0.783 ± 0.01 [b]	52.6 ± 0.001 [cba]
	MSD			4.92	7.64	0.04	2.95
FD	0	3.28 ± 0.019 [a]	0.44 ± 0.024 [a]	74.05 ± 5.19 [a]	28.86 ± 1.48 [a]	0.893 ± 0.03 [a]	40.6 ± 0.001 [a]
	30 days	3.57 ± 0.012 [a]	0.43 ± 0.024 [a]	63.92 ± 5.73 [b]	32.97 ± 0.85 [a]	0.913 ±0.01 [a]	40.3 ± 0.001 [a]
	90 days	3.47 ± 0.055 [a]	0.44 ± 0.024 [a]	54.97 ± 5.60 [b]	35.65 ± 2.19 [a]	0.893±0.03 [a]	41.3 ± 0.002 [a]
	120 days	3.49 ± 0.032 [a]	0.38 ± 0.008 [a]	40.86 ± 2.99 [c]	32.01 ± 1.49 [a]	0.911±0.00 [a]	41.1 ± 0.001 [a]
	MSD			9.18			

Note: the same letter in the same column means that there is no difference between the samples at the 5% significance level. Source: Authors. MSD: minimal significant difference. Means of the three repetitions of the physicochemical analyses ±standard deviations. Means with the same letter in the same column for the same jam do not differ significantly from each other (α = 5%). FA: conventional jam prepared with Powder Blue fruit and synthetic ATM pectin. FB: light jelly prepared with fruit of the Powder Blue variety and pectin BTM+ gummies. FC: conventional jelly prepared with Climax fruit and pectin extracted from passion fruit peel. FD: light jelly prepared with fruit from the Climax variety and pectin BTM+ gummies.

Table S2. Texture analysis results of Climax and Powder blue blueberry jellies.

Property	Storage (Days)	FA	FB	FC	FD
Hard-ness (N)	0	11.06 ± 1.13 [a]	6.24 ± 0.48 [a]	6.99 ± 0.05 [a]	5.92 ± 0.38 [a] (N)
	30	11.58 ± 1.34 [a]	5.88 ± 0.212 [a]	7.00 ± 0.06 [a]	5.90 ± 0.12 [a] (N)
	90	9.36 ± 0.50 [b]	4.37 ± 0.25 [b]	7.06 ± 0.04 [a]	4.83 ± 0.56 [b] (N)
	120	8.09 ± 0.62 [b]	4.51 ± 0.33 [b]	6.89 ± 0.07 [a]	4.86 ± 0.35 [b] (N)
MSD		2.19	0.77		0.84
Adhesiveness (mJ)	0	84.54 ± 2.85 [a]	69.75 ± 3.52 [a]	38.74 ± 3.00 [a]	37.83 ± 2.58 [a]
	30	73.04 ± 3.78 [b]	59.96 ± 3.73 [b]	24.51 ± 3.26 [b]	22.91 ± 2.17 [b]
	90	28.14 ± 4.88 [c]	42.32 ± 2.68 [c]	20.56 ± 1.99 [b]	25.61 ± 2.08 [b]
	120	19.6 ± 2.15 [d]	39.54 ± 2.54 [c]	18.77 ± 1.78 [b]	20.86 ± 2.19 [b]
MSD		8.32	8.28	6.58	5.58

Note: the same letter in the same column means that there is no difference between the samples at the 5% significance level.

Table S3. Weights and bias of the optimized ANN architecture for the Input—First Hidden layer connections.

j	Weights										Bias
	w^I_{j1}	w^I_{j2}	w^I_{j3}	w^I_{j4}	w^I_{j5}	w^I_{j6}	w^I_{j7}	w^I_{j8}	w^I_{j9}	w^I_{j10}	$b^{H1}{}_j$
1	−0.6357	−0.5108	−0.1786	0.5038	−0.3848	−1.0681	0.4206	−0.7459	2.0406	−0.3282	−0.5309
2	−1.0896	0.3475	0.6895	0.3261	−0.4256	0.5366	0.2124	0.4905	−0.6655	0.2671	−0.9347
3	−0.7812	0.6989	0.2713	−0.4453	−0.2633	−0.4535	0.7429	0.6091	−0.3293	0.2657	−0.7290
4	0.7032	−0.6687	0.3930	0.0865	0.5853	−0.3894	−0.3574	−0.6595	−0.7117	2.0604	0.3068
5	0.4649	−0.6013	−0.1172	0.1648	0.5654	0.0815	0.7869	0.0528	0.5512	−0.7455	0.6457
6	−0.5261	0.2816	−0.6346	0.1892	−0.4504	3.0662	0.7709	0.2412	0.5332	−0.7967	−1.1694
7	−0.3938	−3.0081	0.3432	0.3051	0.5848	0.4533	−0.1817	0.3976	−0.4579	1.0286	0.7389
8	0.2551	−0.1464	−0.5807	0.3040	0.6392	0.4095	0.1962	−0.3702	0.4444	−0.0924	−0.8233
9	−0.3732	0.7073	0.5536	0.3318	−0.6542	0.7022	0.7249	−0.4923	−0.2418	−0.4644	−0.1817
10	−0.1013	0.7471	1.0960	0.1323	0.3160	0.0486	0.3314	−0.0697	0.1553	0.3933	0.0036
11	−0.4934	−0.7023	−0.0342	0.6094	0.0614	2.3166	0.0935	0.6375	−0.5524	0.2920	0.9965
12	−0.1436	−0.6840	−0.4256	−0.3347	0.1771	0.7670	0.5477	−0.0755	−0.3801	−0.7056	−0.6661
13	0.1976	0.0851	−0.5886	−0.5298	0.1291	−0.1918	−0.3245	0.2479	−0.4210	−2.1438	−1.1635
14	−0.3553	−0.6451	0.4384	−0.4536	0.3032	0.0459	0.4028	−0.6441	0.7233	−0.1907	0.4716
15	−0.3040	0.1492	0.2667	0.4454	−0.4185	0.3459	−0.4031	−0.1360	−0.1285	−0.6705	0.5405
16	−0.1416	−0.0720	0.2452	0.5479	−0.5831	0.0629	0.4834	0.5011	0.6442	−0.4323	−0.0211
17	−0.7250	0.7296	−0.1576	0.5177	1.0493	−0.7753	−0.3572	−0.4582	0.2340	1.5165	0.3665
18	−0.3520	−1.2704	−0.7613	−0.1229	0.6842	−0.1499	0.1523	−2.0210	−0.7409	−0.3255	0.3658
19	0.5149	−0.3997	0.6444	0.1653	−0.0250	−0.1741	0.1626	−0.3129	−0.4441	0.3424	0.8492
20	−0.2814	−0.2751	0.1773	−0.5971	−0.1645	0.3689	0.1572	−0.6257	−0.1560	−0.3506	0.8369
21	−0.3793	0.3922	−0.1040	−0.0682	−0.3888	−0.4364	0.4193	−0.4344	−0.0210	−0.7604	−0.8171
22	−0.3954	0.2263	−0.4532	−0.2175	0.5788	0.3323	−0.5057	−0.7909	−0.1237	−0.5986	−1.1115
23	−0.7860	−0.2951	−0.4913	2.1674	0.3150	0.2264	−1.7108	−0.2315	−0.0076	−0.0204	0.2746
24	0.3712	−0.7540	−1.0952	−0.3667	−0.6334	−0.6670	0.1721	−0.0207	0.7810	0.0025	−0.5019
25	0.6271	−0.7043	−0.0533	0.7517	0.6452	1.0558	0.7987	−0.6721	−0.3424	−0.4126	−0.2957
26	−0.1059	−0.4427	2.0445	0.9746	0.6998	0.3581	0.5040	0.4151	−0.1502	3.0078	0.6785
27	−0.0913	−1.6201	0.3687	−0.4960	−0.0222	−0.7863	0.0864	−0.3973	−0.0912	−0.1354	−1.1403
28	0.4337	−0.7782	−0.0126	−1.0290	0.3726	0.3009	−2.0360	0.4266	−0.3380	−0.6366	−0.8983
29	−0.2684	0.7392	−0.4331	−0.7450	0.6544	−0.6694	0.4604	0.6413	−0.7054	0.4592	0.3591
30	0.5916	1.6489	0.3135	0.6132	0.6492	−0.1005	1.5804	0.7683	−0.3985	0.2320	−1.1271

Table S4. Weights and bias of the optimized ANN architecture for the First Hidden layer—Second Hidden layer connections.

j	Weights										Bias
	w^{H1-H2}_{j1}	w^{H1-H2}_{j2}	w^{H1-H2}_{j3}	w^{H1-H2}_{j4}	w^{H1-H2}_{j5}	w^{H1-H2}_{j6}	w^{H1-H2}_{j7}	w^{H1-H2}_{j8}	w^{H1-H2}_{j9}	w^{H1-H2}_{j10}	b^{H1-H2}_{j}
1	−2.6449	1.3735	−0.9075	4.1159	2.9453	4.8557	−4.8126	−1.6643	4.3173	−5.8680	2.5017
2	−4.8824	1.6616	5.7123	3.0834	−5.4991	−2.1752	4.3592	−5.6295	−2.8701	−1.3992	2.7613
3	−1.9045	2.9827	−2.1777	−3.0621	−5.1626	4.2623	−3.6795	−3.6362	3.5036	3.8335	−2.1512
4	4.8963	1.5703	1.9533	0.0096	4.0637	0.0364	4.8571	1.8921	5.7883	−5.8431	1.7418
5	−0.9085	5.2861	−4.3578	−0.6933	−3.7329	1.4681	5.9214	4.3376	1.5197	−1.2636	1.9046
6	3.6961	3.0207	−5.0122	4.0037	−4.3503	2.5647	−3.4917	−3.2834	−1.5362	−3.2114	−1.9628
7	3.9373	−3.4505	−2.1303	3.8368	−0.0744	0.9233	−3.6643	1.0608	−3.2109	3.8113	0.8077
8	2.3162	−3.2266	4.5751	−1.9204	1.2481	1.3031	−1.9635	−0.2163	5.0436	−5.9167	0.8956
9	−3.8509	2.9992	1.3154	−3.7692	0.5952	−0.9935	−2.0661	−5.9197	−2.6953	2.4069	1.7416
10	−3.4843	3.0664	0.8155	3.3501	5.6696	4.3377	−5.6117	−5.6110	−2.2471	2.8333	−1.6305

Table S4. (continuation).

J	Weights										Bias
	w^{H1-H2}_{j11}	w^{H1-H2}_{j12}	w^{H1-H2}_{j13}	w^{H1-H2}_{j14}	w^{H1-H2}_{j15}	w^{H1-H2}_{j16}	w^{H1-H2}_{j17}	w^{H1-H2}_{j18}	w^{H1-H2}_{j19}	w^{H1-H2}_{j20}	b^{H1-H2}_{j}
1	4.2187	−3.9299	−5.5090	5.1692	−0.8806	−4.5731	2.0447	1.8047	2.3807	4.6724	2.1841
2	3.6943	−3.7065	4.7034	0.9581	−5.7107	2.0814	2.2893	−0.0373	−3.7324	0.6655	3.1063
3	4.8175	1.6356	1.2392	−5.4923	−1.8963	−0.6420	−5.9814	5.4615	1.6483	−0.4983	−3.3512
4	4.4362	−2.8753	−0.8116	−2.2844	−5.9524	1.0622	2.1006	1.0702	4.8241	2.3121	2.7151
5	−2.2705	5.4765	2.5333	0.5222	2.3211	1.5264	5.9252	−1.3352	−2.3025	−1.7018	−1.6329
6	−5.0202	5.95107	2.5484	1.8481	0.9783	3.5185	−1.7921	−2.5298	−2.4702	0.8699	1.0849
7	−1.4195	−5.0702	−0.9431	0.1597	0.2646	−5.3451	2.1795	1.7123	−0.0853	0.4415	0.8163
8	5.1287	−3.2313	−1.9736	−3.5944	−4.9863	−0.7293	−3.9198	−4.0792	0.8218	0.9197	1.8417
9	2.1621	2.6651	3.4643	0.1847	−1.2464	−4.5309	−3.1384	−0.4014	4.7614	−3.1643	−0.7457
10	−0.7538	−1.7775	3.2508	1.4081	3.4759	−1.3006	−5.5583	−0.1982	−0.4537	4.8526	0.6978

Table S4. (continuation).

j	Weights										Bias
	w^{H1-H2}_{j21}	w^{H1-H2}_{j22}	w^{H1-H2}_{j23}	w^{H1-H2}_{j24}	w^{H1-H2}_{j25}	w^{H1-H2}_{j26}	w^{H1-H2}_{j27}	w^{H1-H2}_{j28}	w^{H1-H2}_{j29}	w^{H1-H2}_{j30}	b^{H1-H2}_{j}
1	−1.2324	0.7433	−0.8924	−2.5185	−0.2917	3.9491	−2.0469	−1.5108	−3.5564	−5.4118	2.8581
2	2.7317	2.8182	4.8312	−5.3062	2.7193	−3.8453	−5.4573	−0.8083	0.3001	1.7973	3.6352
3	−0.8791	−4.8031	−3.8428	5.9568	−1.3651	−4.2402	4.6511	1.8695	0.0503	2.5621	−3.9145
4	4.2782	−5.2326	−3.8824	2.5483	−0.1343	−3.9847	0.4746	3.2442	0.9205	0.1083	1.7419
5	−5.5093	−5.9564	2.6671	−0.9651	5.9928	5.1745	1.5572	−2.4114	−1.0794	4.8402	−2.6235
6	0.2847	1.0872	−4.8034	−1.4655	0.3944	−2.1708	−4.0131	−3.6701	4.7491	4.7127	1.6081
7	4.6031	3.1148	0.3933	2.5438	5.3172	−1.0404	−2.4028	0.4847	−5.7228	5.8456	−1.4051
8	−1.7988	3.3715	−2.7318	−0.7723	0.6027	2.4671	−1.2503	−0.0233	−3.1493	2.0214	1.3665
9	−3.8274	−1.4439	2.7892	3.6589	−0.5113	−1.4414	4.9486	−3.0428	−4.4251	0.3976	0.8048
10	−4.1866	2.9951	0.1116	−1.1921	−5.0384	0.9771	−4.2623	−0.0769	−4.6909	−0.2625	0.6307

Table S5. Weights and bias of the optimized ANN architecture for the Second Hidden layer—Output connections.

j	Weights										Bias
	w^{H2}_{j1}	w^{H2}_{j2}	w^{H2}_{j3}	w^{H2}_{j4}	w^{H2}_{j5}	w^{H2}_{j6}	w^{H2}_{j7}	w^{H2}_{j8}	w^{H2}_{j9}	w^{H2}_{j10}	b^{0}_{k}
1	−0.2641	0.5178	0.1109	−0.8409	0.8597	−0.7761	−0.9684	−0.3583	−0.7109	0.3125	−0.8085
2	−0.6923	−0.9211	0.0572	0.4562	−0.1613	−0.0598	−0.7016	−0.0947	0.9221	0.2533	−0.7813
3	0.7614	−0.5830	0.4823	−0.5695	−0.1327	0.9143	0.5075	0.3591	−0.4193	−0.5674	−1.0418

Table S6. Results obtained for the three dependent variables for the prediction step for architectures X-X-3.

Architecture	Run	% Hardness	% Water Activity	% Adhesiveness	% Average
2-3-3	26	0.55	0.90	0.68	0.71
2-5-3	29	1.14	0.89	0.73	0.92
2-10-3	9	0.94	0.60	0.90	0.81
2-15-3	50	0.54	0.88	0.58	0.67
2-20-3	16	1.07	0.51	1.09	0.89
2-25-3	43	0.87	1.00	0.93	0.93
2-30-3	13	1.17	1.17	1.09	1.14
3-3-3	11	1.20	1.10	0.87	1.06
3-5-3	25	0.50	1.01	1.20	0.90
3-10-3	40	0.92	1.10	0.56	0.86
3-15-3	44	0.89	1.19	0.55	0.88
3-20-3	23	0.78	0.61	0.97	0.79
3-25-3	15	1.14	1.10	1.16	1.13
3-30-3	22	0.91	0.72	0.87	0.83
5-3-3	24	0.64	0.87	1.06	0.86
5-5-3	7	1.00	0.60	1.06	0.89
5-10-3	29	1.14	0.87	0.86	0.96
5-15-3	2	0.97	0.81	0.55	0.78
5-20-3	25	1.12	0.95	0.62	0.90
5-25-3	9	0.74	0.87	0.73	0.78
5-30-3	17	0.71	0.94	1.08	0.91
10-3-3	12	1.10	0.86	1.02	0.99
10-5-3	37	1.19	0.60	0.97	0.92
10-10-3	15	0.69	0.70	0.55	0.65
10-15-3	2	0.98	1.06	0.73	0.92
10-20-3	46	0.58	1.19	1.10	0.96
10-25-3	9	0.64	0.51	1.09	0.75
10-30-3	40	1.12	1.17	0.63	0.97
15-3-3	28	0.67	1.14	0.71	0.84
15-5-3	12	1.05	0.98	0.99	1.01
15-10-3	42	1.18	1.19	0.53	0.97
15-15-3	18	1.10	0.75	0.81	0.89
15-20-3	20	0.57	0.73	1.10	0.80
15-25-3	2	1.04	0.70	0.92	0.89
15-30-3	20	0.75	0.56	0.74	0.68
20-3-3	22	0.89	0.55	1.19	0.88
20-5-3	24	1.20	0.93	0.66	0.93
20-10-3	14	0.82	0.75	0.76	0.78
20-15-3	35	0.99	0.80	0.87	0.89
20-20-3	4	0.57	0.87	1.03	0.82
20-25-3	26	0.79	0.61	0.66	0.69
20-30-3	10	0.59	0.58	1.19	0.79
25-3-3	25	1.04	1.04	0.68	0.92
25-5-3	41	1.07	0.83	1.09	1.00
25-10-3	15	0.93	0.80	0.50	0.74
25-15-3	16	0.54	0.88	1.20	0.87
25-20-3	28	0.67	0.88	0.58	0.71
25-25-3	18	0.94	0.78	1.07	0.93
25-30-3	35	1.20	1.11	0.57	0.96

Table S7. Results obtained for the three dependent variables for the prediction step for architectures 3-X-X-3.

Architecture	Run	% Hardness	% Water Activity	% Adhesiveness	% Average
3-2-3-3	24	0.81	0.78	0.80	0.80
3-2-5-3	43	0.45	0.46	0.75	0.55
3-2-10-3	12	0.87	0.41	0.27	0.52
3-2-15-3	34	0.39	0.95	0.49	0.61
3-2-20-3	12	0.44	0.81	0.69	0.65
3-2-25-3	4	1.00	0.33	0.84	0.72
3-2-30-3	27	0.44	0.32	0.56	0.44
3-3-3-3	15	0.53	0.66	0.43	0.54
3-3-5-3	2	0.31	0.81	0.87	0.66
3-3-10-3	18	0.93	0.40	0.28	0.54
3-3-15-3	45	0.74	0.93	0.25	0.64
3-3-20-3	20	0.72	0.87	0.44	0.68
3-3-25-3	6	0.91	0.34	0.53	0.59
3-3-30-3	43	0.46	0.86	0.55	0.62
3-5-3-3	15	0.73	0.27	0.74	0.58
3-5-5-3	35	0.43	0.42	0.91	0.59
3-5-10-3	39	0.64	0.54	0.84	0.67
3-5-15-3	3	0.34	0.72	0.97	0.68
3-5-20-3	16	0.85	0.79	0.43	0.69
3-5-25-3	32	0.34	0.77	0.51	0.54
3-5-30-3	36	0.88	0.46	0.67	0.67
3-10-3-3	10	0.87	0.30	0.76	0.64
3-10-5-3	13	0.36	0.68	0.65	0.56
3-10-10-3	40	0.81	0.44	0.93	0.73
3-10-15-3	34	0.87	0.35	0.83	0.68
3-10-20-3	15	0.32	0.74	0.28	0.45
3-10-25-3	33	0.70	1.00	0.38	0.69
3-10-30-3	32	0.84	0.78	0.34	0.65
3-15-3-3	27	0.46	0.46	0.86	0.59
3-15-5-3	18	0.53	0.98	0.41	0.64
3-15-10-3	22	0.57	0.81	0.47	0.62
3-15-15-3	43	0.39	0.82	0.88	0.70
3-15-20-3	39	0.53	0.69	0.56	0.59
3-15-25-3	17	0.35	0.52	0.76	0.54
3-15-30-3	32	0.74	0.60	0.55	0.63
3-20-3-3	50	0.68	0.73	0.53	0.65
3-20-5-3	37	0.37	0.31	0.70	0.46
3-20-10-3	50	0.73	0.25	0.67	0.55
3-20-15-3	10	0.87	0.33	0.81	0.67
3-20-20-3	26	0.98	0.92	0.74	0.88
3-20-25-3	21	0.31	0.58	0.95	0.61
3-20-30-3	44	1.00	0.85	0.28	0.71
3-25-3-3	19	0.75	0.25	0.54	0.51
3-25-5-3	29	0.82	0.56	0.49	0.62
3-25-10-3	15	0.62	0.29	0.66	0.52
3-25-15-3	17	0.40	0.68	0.97	0.68
3-25-20-3	22	0.49	0.93	0.83	0.75
3-25-25-3	3	0.67	0.39	0.75	0.60
3-25-30-3	33	0.74	0.40	1.00	0.71

Table S8. Results obtained for the three dependent variables for the prediction step for architectures 5-X-X-3.

Architecture	Run	% Hardness	% Water Activity	% Adhesiveness	% Average
5-3-5-3	42	0.51	0.44	0.41	0.45
5-3-10-3	10	0.28	0.55	0.90	0.58
5-3-15-3	25	0.69	0.57	0.84	0.70
5-3-20-3	36	0.65	0.47	0.40	0.51
5-3-25-3	10	0.73	0.93	0.88	0.85
5-3-30-3	28	0.68	0.50	0.29	0.49
5-5-5-3	1	0.93	0.39	0.40	0.57
5-5-10-3	14	1.00	0.94	0.74	0.89
5-5-15-3	50	0.56	0.27	0.37	0.40
5-5-20-3	8	0.63	0.36	0.95	0.65
5-5-25-3	24	0.53	0.95	0.97	0.82
5-5-30-3	42	0.64	0.43	0.37	0.48
5-10-5-3	15	0.95	0.93	0.61	0.83
5-10-10-3	1	0.46	0.57	0.84	0.62
5-10-15-3	46	0.29	0.31	0.60	0.40
5-10-20-3	26	0.43	0.58	0.59	0.53
5-10-25-3	49	0.99	0.74	0.26	0.66
5-10-30-3	7	0.63	0.67	0.75	0.68
5-15-5-3	33	0.27	0.81	0.72	0.60
5-15-10-3	27	0.52	0.58	0.29	0.46
5-15-15-3	41	0.65	0.81	0.30	0.59
5-15-20-3	38	0.43	0.75	0.99	0.72
5-15-25-3	42	0.89	0.61	0.88	0.79
5-15-30-3	22	0.36	0.78	0.66	0.60
5-20-5-3	1	0.63	0.65	0.98	0.75
5-20-10-3	1	0.67	0.71	0.33	0.57
5-20-15-3	29	0.43	0.90	0.82	0.72
5-20-20-3	12	0.86	0.28	0.74	0.63
5-20-25-3	12	0.96	0.40	0.41	0.59
5-20-30-3	14	0.95	0.60	0.25	0.60
5-25-5-3	8	0.61	0.96	0.66	0.74
5-25-10-3	16	0.71	0.96	0.94	0.87
5-25-15-3	2	0.74	0.53	0.71	0.66
5-25-20-3	26	0.72	0.37	0.58	0.56
5-25-25-3	8	0.28	0.92	0.38	0.53
5-25-30-3	18	0.25	0.48	0.44	0.39
5-30-5-3	40	0.56	0.77	0.54	0.62
5-30-10-3	3	0.32	0.99	0.73	0.68
5-30-15-3	18	0.62	0.80	0.54	0.65
5-30-20-3	10	0.54	0.76	0.77	0.69
5-30-25-3	26	0.62	0.97	0.50	0.70
5-30-30-3	3	0.53	0.92	0.87	0.77

Table S9. Results obtained for the three dependent variables for the prediction step for architectures 7-X-X-3.

Architecture	Run	% Hardness	% Water Activity	% Adhesiveness	% Average
7-3-5-3	26	0.93	0.46	0.92	0.77
7-3-10-3	37	0.94	0.78	0.16	0.63
7-3-15-3	2	0.46	0.73	0.69	0.63
7-3-20-3	34	0.32	0.94	0.44	0.57
7-3-25-3	15	1.00	0.89	0.20	0.70
7-3-30-3	14	0.63	0.56	0.92	0.70
7-5-5-3	38	0.38	0.55	0.77	0.57
7-5-10-3	8	0.40	0.79	0.96	0.72
7-5-15-3	3	1.00	0.46	0.37	0.61
7-5-20-3	9	0.29	0.95	0.43	0.56
7-5-25-3	23	0.25	0.92	0.84	0.67
7-5-30-3	5	1.00	0.54	0.94	0.83
7-10-5-3	8	0.87	0.84	0.64	0.78
7-10-10-3	20	0.89	0.75	0.49	0.71
7-10-15-3	18	0.45	0.37	0.31	0.38
7-10-20-3	36	0.19	0.40	0.15	0.25
7-10-25-3	40	0.73	0.93	0.20	0.62
7-10-30-3	15	0.18	0.31	0.33	0.27
7-15-5-3	13	1.00	0.50	0.55	0.68
7-15-10-3	20	0.42	0.44	0.56	0.47
7-15-15-3	49	0.16	0.34	0.55	0.35
7-15-20-3	6	0.34	0.85	0.52	0.57
7-15-25-3	47	0.31	0.22	0.79	0.44
7-15-30-3	31	0.30	0.37	0.88	0.52
7-20-5-3	6	0.57	0.69	0.28	0.51
7-20-10-3	14	0.21	0.79	0.39	0.46
7-20-15-3	13	0.49	0.16	0.27	0.31
7-20-20-3	24	0.45	0.81	0.45	0.57
7-20-25-3	6	0.86	0.96	0.50	0.77
7-20-30-3	14	0.34	0.23	0.58	0.38
7-25-5-3	17	0.51	0.21	0.30	0.34
7-25-10-3	3	0.84	0.72	0.67	0.74
7-25-15-3	37	0.95	0.62	0.99	0.85
7-25-20-3	29	0.26	0.61	0.35	0.41
7-25-25-3	13	0.76	0.45	0.66	0.62
7-25-30-3	16	0.88	0.35	0.58	0.60
7-30-5-3	15	0.72	0.90	0.62	0.75
7-30-10-3	16	0.68	0.45	0.84	0.66
7-30-15-3	45	0.71	0.57	0.25	0.51
7-30-20-3	32	0.59	0.43	0.39	0.47
7-30-25-3	18	0.62	0.36	0.68	0.55
7-30-30-3	50	0.67	0.42	0.33	0.47

Table S10. Results obtained for the three dependent variables for the prediction step for architectures 10-X-X-3.

Architecture	Run	% Hardness	% Water Activity	% Adhesiveness	% Average
10-3-5-3	44	0.22	0.11	0.45	0.26
10-3-10-3	22	0.14	0.08	0.32	0.18
10-3-15-3	27	0.25	0.11	0.55	0.30
10-3-20-3	23	0.09	0.05	0.28	0.14
10-3-25-3	15	0.23	0.07	0.32	0.21
10-3-30-3	32	0.15	0.05	0.24	0.15
10-5-5-3	21	0.17	0.06	0.29	0.17
10-5-10-3	32	0.35	0.16	1.40	0.64
10-5-15-3	21	0.22	0.05	0.25	0.17
10-5-20-3	18	0.27	0.03	0.33	0.21
10-5-25-3	27	0.16	0.08	0.18	0.14
10-5-30-3	35	0.13	0.03	0.34	0.17
10-10-5-3	26	0.15	0.04	0.23	0.14
10-10-10-3	8	0.36	0.10	0.40	0.29
10-10-15-3	6	0.31	0.09	0.30	0.23
10-10-20-3	12	0.19	0.05	0.24	0.16
10-10-25-3	1	0.22	0.10	0.29	0.20
10-10-30-3	42	0.25	0.07	0.29	0.20
10-15-5-3	48	0.15	0.05	0.47	0.22
10-15-10-3	9	0.14	0.08	0.36	0.19
10-15-15-3	7	0.43	0.13	0.50	0.35
10-15-20-3	34	0.18	0.17	0.38	0.24
10-15-25-3	44	0.59	0.06	0.52	0.39
10-15-30-3	49	0.19	0.08	0.36	0.21
10-20-5-3	3	0.33	0.08	0.26	0.22
10-20-10-3	4	0.13	0.05	0.30	0.16
10-20-15-3	12	0.24	0.04	0.89	0.39
10-20-20-3	21	0.19	0.03	0.41	0.21
10-20-25-3	16	0.20	0.06	0.59	0.28
10-20-30-3	27	0.34	0.06	0.42	0.27
10-25-5-3	30	0.62	0.22	0.16	0.33
10-25-10-3	46	0.30	0.06	0.27	0.21
10-25-15-3	47	0.13	0.08	0.36	0.19
10-25-20-3	29	0.22	0.12	0.51	0.28
10-25-25-3	40	0.14	0.06	0.14	0.11
10-25-30-3	27	0.36	0.10	0.59	0.35
10-30-5-3	28	0.23	0.07	0.50	0.27
10-30-10-3	44	0.06	0.07	0.13	0.09
10-30-15-3	3	0.14	0.08	0.13	0.12
10-30-20-3	42	0.37	0.13	0.21	0.24
10-30-25-3	31	0.22	0.05	0.36	0.21
10-30-30-3	30	0.11	0.06	0.57	0.25

Table S11. Results obtained for the three dependent variables for the prediction step for architectures 15-X-X-3.

Architecture	Run	% Hardness	% Water Activity	% Adhesiveness	% Average
15-3-5-3	20	0.74	0.85	0.14	0.58
15-3-10-3	45	0.43	0.21	0.05	0.23
15-3-15-3	1	0.42	0.27	0.65	0.45
15-3-20-3	31	0.31	0.78	0.40	0.50
15-3-25-3	33	0.79	0.73	0.60	0.71
15-3-30-3	37	0.87	0.94	0.21	0.67
15-5-5-3	13	0.24	0.54	0.61	0.46
15-5-10-3	19	0.92	0.77	0.32	0.67
15-5-15-3	39	0.84	0.28	0.11	0.41
15-5-20-3	38	0.37	0.61	1.00	0.66
15-5-25-3	30	0.26	0.75	0.90	0.64
15-5-30-3	46	0.90	0.98	0.98	0.95
15-10-5-3	26	0.71	0.94	0.49	0.71
15-10-10-3	27	0.98	0.69	0.60	0.76
15-10-15-3	12	0.36	0.52	0.63	0.50
15-10-20-3	8	0.02	0.12	0.71	0.28
15-10-25-3	32	0.85	0.31	0.36	0.51
15-10-30-3	7	0.80	0.58	0.89	0.76
15-15-5-3	20	0.24	0.66	0.32	0.41
15-15-10-3	31	0.88	0.47	0.05	0.47
15-15-15-3	11	0.26	0.68	0.46	0.47
15-15-20-3	38	0.67	0.82	0.77	0.75
15-15-25-3	49	0.88	0.69	0.83	0.80
15-15-30-3	46	0.83	0.98	0.79	0.87
15-20-5-3	20	0.62	0.66	0.51	0.60
15-20-10-3	39	0.40	0.73	0.58	0.57
15-20-15-3	33	0.40	0.19	0.97	0.52
15-20-20-3	4	0.54	0.91	0.73	0.73
15-20-25-3	44	0.57	0.57	0.38	0.51
15-20-30-3	42	0.78	0.97	0.76	0.84
15-25-5-3	8	0.92	0.39	0.76	0.69
15-25-10-3	18	0.11	0.05	0.18	0.11
15-25-15-3	41	0.45	0.25	0.80	0.50
15-25-20-3	45	0.16	0.62	0.18	0.32
15-25-25-3	47	0.72	0.42	0.80	0.65
15-25-30-3	31	0.31	0.13	0.24	0.23
15-30-5-3	39	0.29	0.93	0.70	0.64
15-30-10-3	33	0.30	0.29	0.77	0.45
15-30-15-3	6	0.10	0.74	0.20	0.35
15-30-20-3	39	0.17	0.73	0.06	0.32
15-30-25-3	12	0.26	0.66	0.64	0.52
15-30-30-3	14	0.99	0.96	0.15	0.70

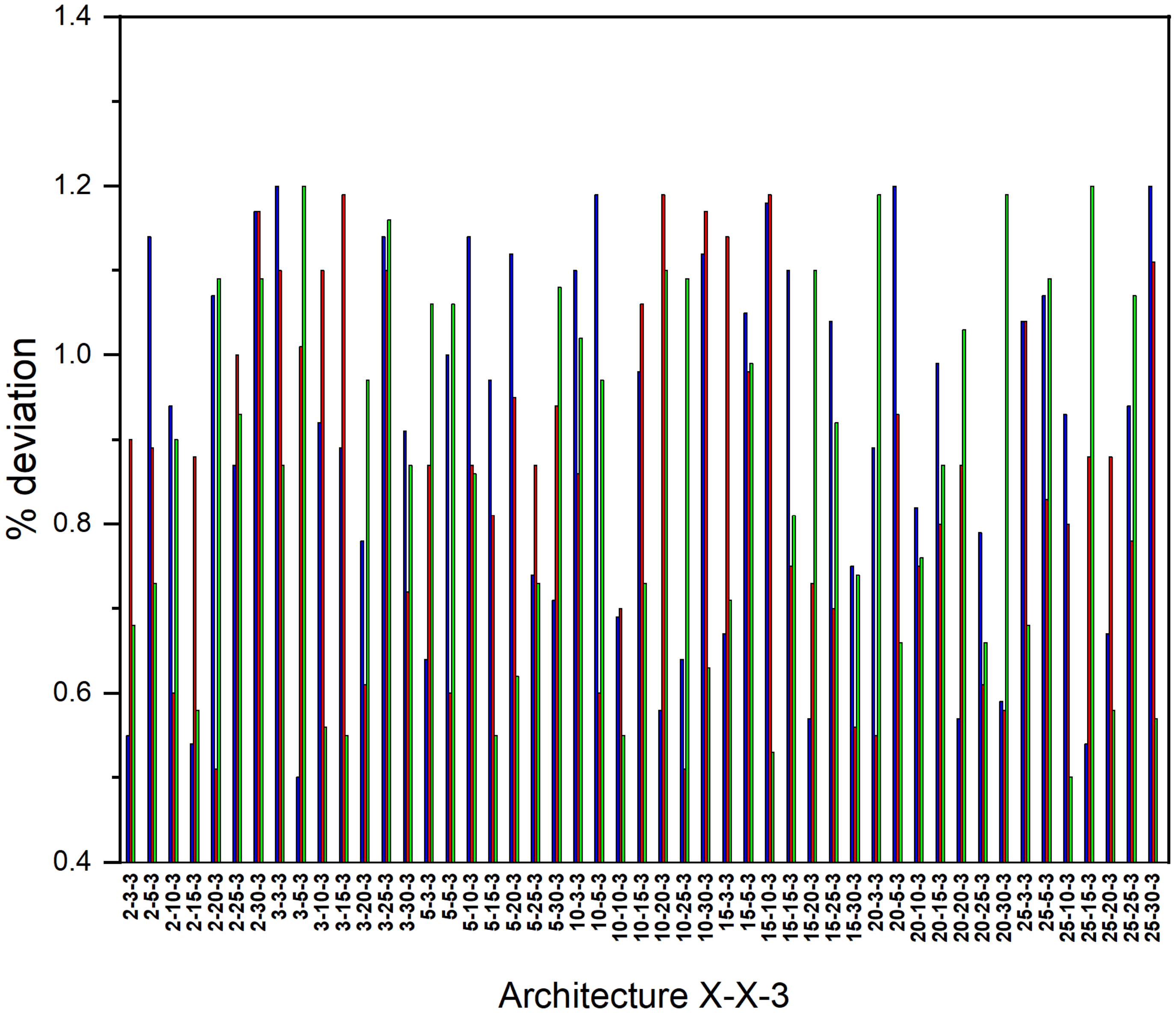

Figure S1. Architecture X-X-3 (blue: % hardness, red: % water activity, green: % adhesiveness).

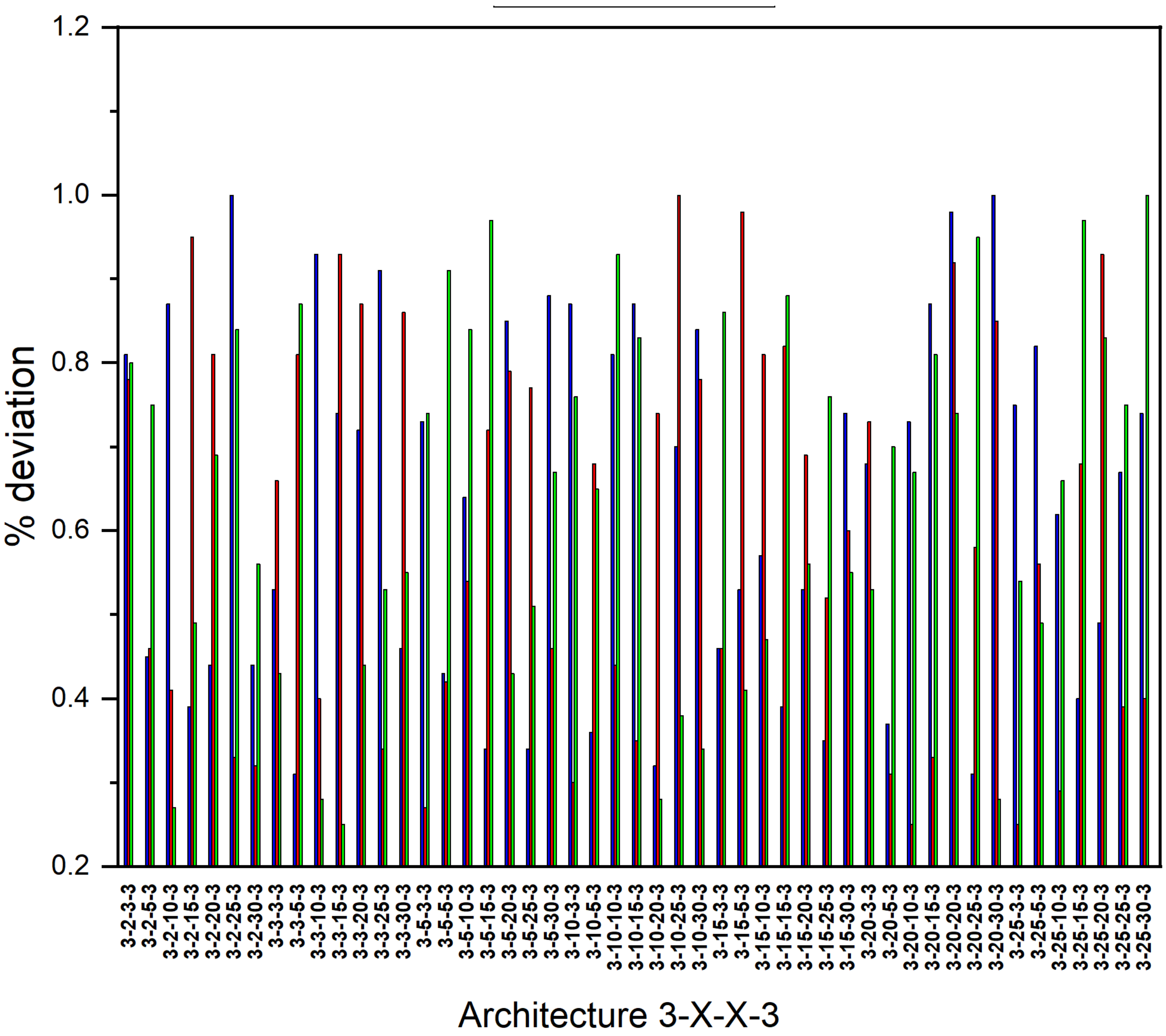

Figure S2. Architecture 3-X-X-3 (blue: % hardness, red: % water activity, green: % adhesiveness).

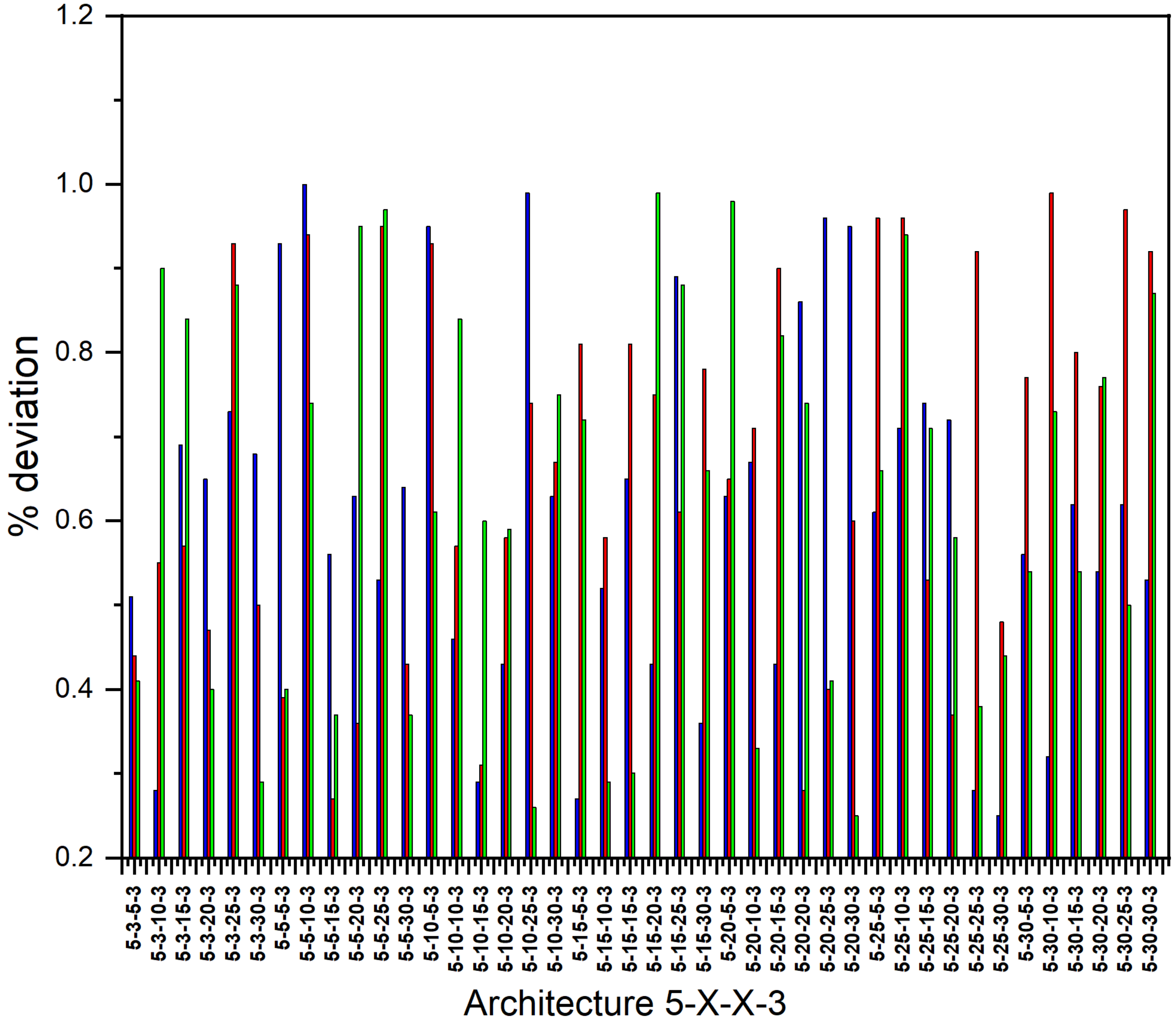

Figure S3. Architecture 5-X-X-3 (blue: % hardness, red: % water activity, green: % adhesiveness).

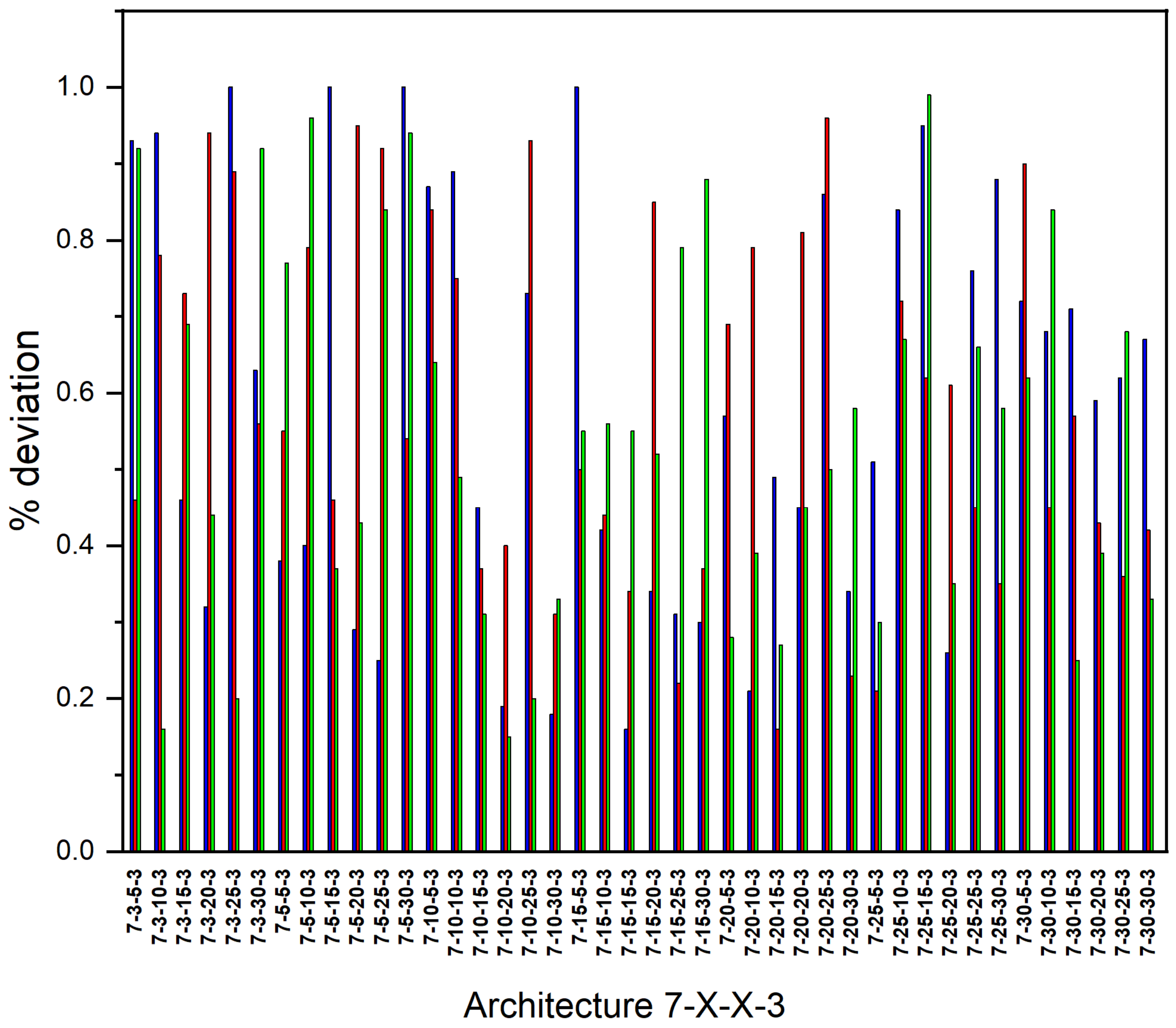

Figure S4. Architecture 7-X-X-3 (blue: % hardness, red: % water activity, green: % adhesiveness).

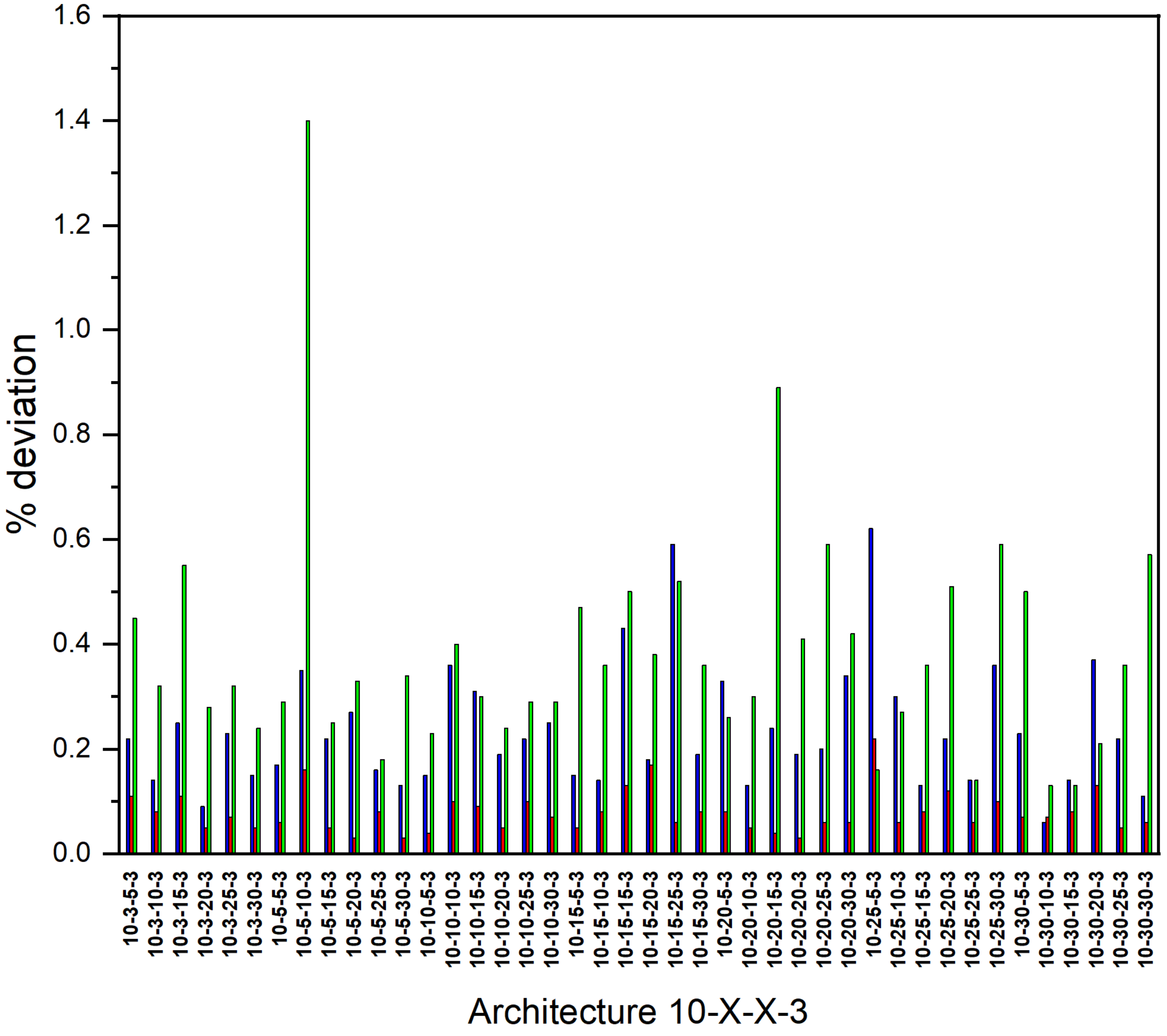

Figure S5. Architecture 10-X-X-3 (blue: % hardness, red: % water activity, green: % adhesiveness).

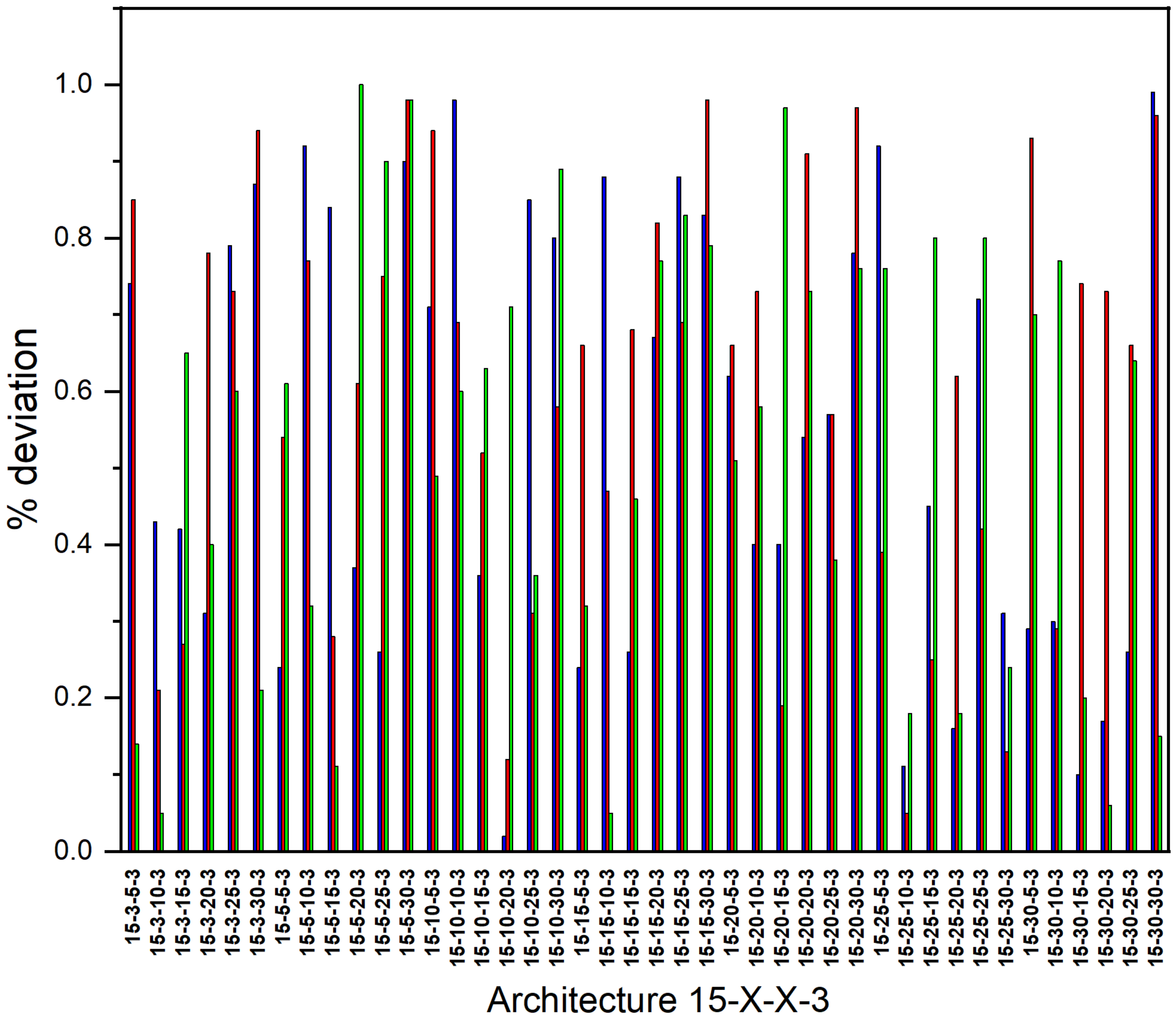

Figure S6. Architecture 15-X-X-3 (blue: % hardness, red: % water activity, green: % adhesiveness).

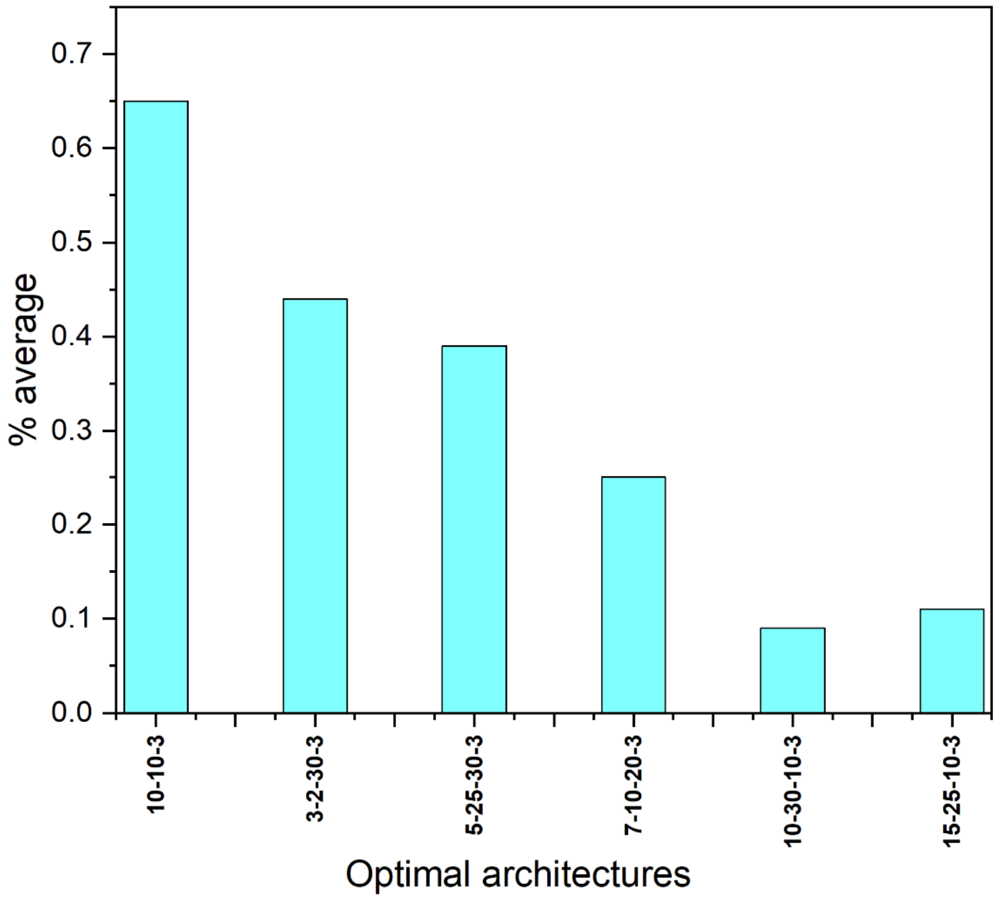

Figure S7. Optimal architectures for three and four layers.
